# Sulfidic toluene mineralization by aquifer microbial communities at different temperatures

**DOI:** 10.1093/femsec/fiaf079

**Published:** 2025-07-29

**Authors:** Mohammad Sufian Bin Hudari, Carsten Vogt

**Affiliations:** Department of Technical Biogeochemistry, Helmholtz Centre for Environmental Research – UFZ, 04318 Leipzig, Permoserstraße 15, Germany; Department of Technical Biogeochemistry, Helmholtz Centre for Environmental Research – UFZ, 04318 Leipzig, Permoserstraße 15, Germany

**Keywords:** high-temperature aquifer thermal energy storage, toluene mineralization, dissimilatory sulfate reduction, microbial community composition, groundwater, stable isotopes

## Abstract

High-temperature aquifer thermal energy storage (HT-ATES) is a carbon-neutral technology in the heating and cooling sector particularly suitable for urban areas, where aquifers are often contaminated with hydrocarbons. How HT-ATES could influence the natural degradation of contaminants such as hydrocarbons has hardly been investigated. Here, we determined the effects of temperature and temperature shifts on the capability of aquifer microbial communities to mineralize the model hydrocarbon toluene at sulfate-reducing conditions. Distinct toluene-mineralizing, sulfate-reducing consortia were enriched from material of two hydrocarbon-contaminated field sites at 12°C, 20°C, 25°C, 38°C, and 45°. Lowest toluene mineralization rates were observed at 38°C, and highest rates were observed at 45°C. Consortia adapted to 12°C or 25°C were generally negatively impacted by temporary or permanent temperature shifts to temperatures ≥ 38°C. *Desulfosporosinus* phylotypes dominated enrichments at 12°C, indicating a major role for toluene mineralization at *in situ* temperatures. At 20°C–25°C, typical sulfate-reducing genera such as *Desulfoprunum, Desulfallas* or *Pelotomaculum* were abundant, indicating synergistic relationships of various toluene degraders belonging to different taxa. The communities grown at 45°C were dominated by putative thermophilic phylotypes affiliated to the phyla *Bacillota* or *Caldiserica*. Overall, our data indicate that 45°C is the upper limit for anaerobic toluene mineralization of the investigated communities.

## Teaser

Distinct toluene-mineralizing, sulfate-reducing consortia were enriched at temperatures between 12°C and 45°C, and were shown to be negatively impacted by temporary or permanent temperature shifts to temperatures ≥ 38°C.

## Introduction

Aquifer thermal energy storage (ATES) is a promising low carbon energy storage technology in the heating and cooling sector (Lee 2010). Currently, 99% of ATES are low-temperature (LT-ATES) installations with operational thresholds of ∼25°C (Fleuchaus et al. 2018, Fleuchaus et al. 2020). Volatile commodity prices have rekindled interest in high-temperature (HT-ATES) that provide higher heat storage capacity (>40°C) and couple as sinks for excess heat generating industries (Fleuchaus et al. 2020). Nevertheless, the environmental impacts, operational costs, and regulatory matters of HT-ATES operations remain unclear (Fleuchaus et al. 2018, Fleuchaus et al. 2020).

ATES are generally constructed in urban and industrial areas with high energy demand that capitalize on their storage and supply capacity. These areas are typically contaminated with anthropogenically derived organic pollutants such as petroleum aromatic hydrocarbons (e.g. benzene-toluene-ethylbenzene-xylene [BTEX]), introduced via leakage or improper disposal (Rooney-Varga et al. 1999, Rittmann et al. 2000, Da Silva and Alvarez 2004, Vogt et al. 2007). In the subsurface, oxygen is rapidly depleted and abiotic processes are limited, hence hydrocarbon degradation is mainly characterized by biotic, anoxic reactions such as dissimilatory microbial sulfate reduction which are slower than aerobic biodegradation processes (Rittmann et al. 2000, Miao et al. 2012, Meckenstock et al. 2015, Lueders 2017). Although anaerobic hydrocarbon degrading microbial consortia and their pathways are well studied, subsurface ecosystems are microbially diverse and heterogeneous with localized niches; hence the site parameters may determine its biotransformation potential (Mancini et al. 2002, McKelvie et al. 2005, Gieg et al. 2010, Winderl et al. 2010, Fuchs et al. 2011, Meckenstock et al. 2015, Lueders 2017, Tian et al. 2017, Garcia et al. 2018, Pannekens et al. 2019).

Besides physicochemical contaminants characteristics, temperature also affects microbial mediated processes, where growth and activity decline rapidly and cease outside optimal environments (Higashioka et al. 2011, Huang et al. 2011, Schipper et al. 2014, Hossain et al. 2017, Madigan et al. 2018, Koproch et al. 2019). Indeed, studies focusing on the effects of increased temperatures on the anaerobic degradation potential of hydrocarbon-impacted aquifer communities are limited; a few showed that the degradation rates increased at higher temperatures, but that too high temperatures (up to 40°C) had an inhibiting effect on degradation (Zeman et al. 2014, Kulkarni et al. 2017). Some studies described how temperature selects for specific communities in habitats with temperature gradients, for example, microbial sulfate reduction was observed in the Guaymas Basin between 3°C and 95°C and in laboratories between 10°C and 102°C, comprising different sulfate reducers with temperature optima of 34°C, 70°C, and 80°C–88°C while hydrocarbon degraders up to about 70°C were enriched (Karl et al. 1988, Elsgaard et al. 1994, Kallmeyer and Boetius 2004, Teske 2024). The current data suggest that the thermal limit for hydrocarbon-degrading microorganisms in hot hydrocarbon reservoirs is between 80°C and 90°C (Aitken et al. 2004, Head et al. 2014, Pannekens et al. 2019). However, with regard to HT-ATES coupled with bioremediation in shallow aquifers where native temperatures are usually far below 30°C, it is currently not known whether thermophilic hydrocarbon-degrading microorganisms are present, or in which numbers, and whether thermophilic hydrocarbon-degrading microorganisms will be active if suitable conditions are created during an HT-ATES operation (Baas Becking and Canfield 2015).

Therefore, in this study, we investigated whether groundwater communities can degrade hydrocarbons at sulfate-reducing conditions at temperatures far above those in their natural habitat. Sulfate is a common electron acceptor in marine and freshwater systems (Jørgensen 1982, Holmer and Storkholm 2001, Zak et al. 2021). Toluene was selected because it is a common aromatic contaminant and well degradable at oxic and anoxic conditions in the mesophilic temperature range (Alvarez et al. 1991, Alvarez and Vogel 1991, Chen et al. 1992, Edwards et al. 1992, Langwaldt and Puhakka 2000, Roychoudhury and McCormick 2006, Roychoudhury and Merrett 2006, Winderl et al. 2008, Meckenstock et al. 2015); moreover, its anaerobic degradation pathway is well understood (Lueders 2017). Degradation of toluene at sulfate-reducing conditions under thermophilic conditions (50–70°C) was reported once (Chen and Taylor 1997), performed by microbial consortia from hot oil reservoirs. Hence, no data are available on the anaerobic degradation potential of aquifer communities at thermophilic conditions from temperate zones where temperatures are permanently moderate. In our study, we selected five different temperatures based on the following rationales: 12°C (native aquifer conditions), 25°C (upper temperature limit for LT-ATES operations in many countries), 38°C (HT-ATES conditions, upper limit for mesophilic conditions), 45°C: (HT-ATES conditions, lower limit for thermophilic conditions), and 60°C (HT-ATES conditions, thermophilic conditions). During HT-ATES operations, the selected temperatures can be attained in the core zone (hottest area) or along a temperature gradient which develops in the surrounding zone (Lerm et al. 2013, Daniilidis et al. 2022). Aquifer microbial communities were obtained from package materials (gravel) derived from a former *in situ-*column experiment at a hydrocarbon-contaminated aquifer (Zt) and from sediments from a hydrocarbon-impacted aquifer (Wg). These were used to set up enrichment cultures at the selected temperatures at sulfate-reducing conditions with toluene as a sole source of carbon and energy. Key players of toluene mineralization at different temperatures were identified by 16S rRNA gene analysis. Active cultures were subjected to temperature changes in additional experiments aiming to simulate different ATES treatment regimes, testing the resilience of the temperature-adapted toluene-mineralizing consortia to temporary or permanent temperature fluctuations.

## Materials and methods

Chemicals were purchased from Sigma-Aldrich (USA), Merck (Germany or Switzerland), and Carl Roth (Germany), unless stated otherwise. [^13^C]-α-toluene was purchased from Sigma-Aldrich (99% purity, 99 atom % at α position, Missouri, USA).

### Culture setup

Initially, seven different enrichment cultures were set-up at several temperatures using gravel from the Zeitz site (Zt) and aquifer sediments from the Weißandt-Gölzau site (Wg) (Fig. S1_Supplementary Data).

#### Set-up of toluene-amended enrichment cultures with Zeitz gravel

Gravel was collected from columns used in an *on-site* percolation installment with sulfidogenic benzene-contaminated groundwater operated for 10 years and 6 months at around 15°C as described elsewhere (Vogt et al. 2007, Taubert et al. 2012). The gravel was obtained in January 2017 when the column experiment was finished and incubated thereafter in the laboratory in 1-L Schott bottles at room temperature (20°C) with anoxic bicarbonate-buffered mineral salt media comprising 10-mM sulfate and 1-mM benzene in 5 mL of the inert carrier phase 2,2,4,4,6,8,8-heptamethylnonane (HMN) and maintained initially as a benzene-amended enrichment culture and constantly replenished with benzene and sulfate. In June 2019, in two bottles, the mineral salt media were discarded, the gravel was mixed in a beaker, 400-g gravel were redistributed each into six 500-mL Schott bottles, and amended each with 300-mL BUS medium (BUS = Benzene Utilizing Sulfate Reducer medium; Table S1_Supplementary Data), supplemented with HMN (5 mL) and 5 µL of toluene, and sealed gas-tight with butyl stoppers fixed with screw caps. The cultures were incubated at six different temperatures (12°C (Zt12), 20°C (Zt20), 25°C (Zt25), 38°C (Zt38), 45°C (Zt45), 60°C (Zt60)) using incubators from the companies Heraeus (Germany, model B6060; for 20°; 25°C and 38°C), Memmert (Germany, model IN30; for 45°C and 60°C) and Binder (Germany, cooling incubator; for 12°C) and maintained for 21 months. Sulfate-reducing activity was monitored by sulfide production. Of the six bottles started, activity could be determined only in Zt12, Zt20, and Zt25 (results not shown).

#### Set-up of toluene-amended enrichment cultures with Weißandt-Gölzau aquifer sediment

Sediments were obtained from a hydrocarbon-contaminated aquifer in Weißandt-Gölzau (51°40′N 12°4′E) in Saxony-Anhalt, Germany, a former site of a brown coal processing industry where sulfate reduction and methanogenesis were considered among the predominant electron accepting processes in the aquifer (Feisthauer et al. 2012). Sediments were collected during a drilling campaign in July 2012, placed in air-tight jars and stored at anoxic conditions at 4°C. In April 2019, enrichment cultures were set up, using sediments sampled from a well at a depth of 8.5–9.5 m. Enrichment cultures were prepared by mixing 3 kg of sediment and 100-mL BUS medium (Table S1_Supplementary Data) in a sterile beaker, subsequent distribution of the resulting slurry (∼510 g each) in five 1-L bottles (Schott, Germany) topped up to around 1 L; each bottle was amended with 5-mL HMN and 5-µL toluene, closed gas-tight with butyl stoppers and fixed with screw caps. The cultures were incubated statically in the dark at five different temperatures, namely, 12°C (Wg12), 25°C (Wg25), 38°C (Wg38), 45°C (Wg45), and 60°C (Wg60), using the same incubators as for the Zt enrichments. Sulfate-reducing activity, determined via sulfide production, was observed in Wg12, WG25, WG38, and WG45 after prolonged incubation. These cultures were further maintained by the addition of toluene (∼ 5 µL, neat) for at least 15 months before proceeding further (see below).

#### Preparation of microcosms used for controlled laboratory experiments

From the active sulfate-reducing enrichment cultures, microcosms for monitoring toluene mineralization were prepared as follows: 5 g of gravel or sediment of a culture was distributed each in a 120-ml serum bottle and around 80-mL BUS medium containing 10-mM sulfate (Table S1_Supplementary Data) was added. The microcosms were sealed gas-tight using butyl septa and aluminum crimps. Prior to toluene addition, all microcosms were incubated at the respective initial temperatures of 12°C, 25°C, or 38°C for three days to condition them at the experimental temperatures before toluene addition. Next, each replicate was amended with 3 mL sterile autoclaved HMN via a plastic syringe (B Braun, Germany) followed by addition of [^13^C]-α-toluene to a final concentration of 1 mM with a glass syringe (Hamilton, USA). Toluene mineralization and sulfate reduction were generally monitored by analyses of δ^13^CO_2_ and sulfide (see below). Thirty-six replicates were each set up with the initial cultures Zt12 (Table S2_Supplementary Data) and Zt25 (Table S3_Supplementary Data), respectively, 45 replicates were set up with each initial cultures Wg12 (Table S4_Supplementary Data), Wg25 (Table S5_Supplementary Data), and Wg38 (Table S6_Supplementary Data). Three replicates were set up for Zt20°C (Zt20), and seven replicates (including one sterile replicate) were set up for Wg45 (Table S7_Supplementary Data). Active enrichment cultures were selected to compare their toluene mineralization and sulfide production rates, and microbial communities at their enrichment temperature. Additional experiments with regard to temperature ‘adaptation’ (cultures exposed permanently to a different temperature) and ‘heat exchange’ (cultures exposed temporarily to a different temperature) were set up using cultures Zt12, Zt25, Wg12, Wg25 and Wg38 to observe short- and long-term effects of temperature stress on temperature-adapted communities.

All microcosms were prepared in a glove box (5% N_2_/5% H_2_ gas atmosphere, Coy Laboratory Products, USA) under anoxic conditions using sterile equipment. All equipment was generally sterilized by autoclaving (121°C, 20 min).

### Chemical analyses

#### *δ^13^*CO_2_  *analyses*

Headspace samples were collected with N_2_-flushed plastic syringes and stored in N_2_-flushed glass vials (5 mL) crimped with Teflon-coated butyl rubber septa and aluminum crimps before analysis. Measurements of CO_2_ and CH_4_ carbon isotope ratios were conducted by gas chromatography-combustion-isotopic ratio mass spectrometry (GC-IRMS) consisting of a gas chromatograph (Agilent, USA) containing a PoraplotQ column (25 m × 0.32 mm ID, 1-µM film; Chrompack, Middleburg, USA) coupled to a mass spectrometer via a Conflow III interface (Germany). Carbon dioxide separation was conducted isothermally at 40°C with helium carrier gas at a flow rate of 2 mL min^−1^. Post-separation products were transferred to a combustion furnace (GC/C-III; Thermo Fisher Scientific, Bremen, Germany) maintained at 980°C on a CuO/Ni/Pt catalyst that also tracks carbon isotope signatures of methane converted to carbon dioxide, although this was not detected in any of the samples. Samples with varying volumes (50–500 µL) were injected at 1:5 split ratios. Carbon isotope signature data are shown in delta notation (per mille, ‰) relative to the Pee Dee Belemnite standard.

The d^13^C/^12^C data in per mille (‰) were used to calculate the extent of mineralization (MIN %) of each replicate using the following equation (Dorer et al. 2016):


(1) \begin{eqnarray*}
{\mathrm{MIN}}\ \left( \% \right) &=& \ \frac{{\left[ {HCO_3^ - } \right] \times \ {{R}_{\textit{VPDB}}}\ \times \ \left( {{{\delta }_{end}} - {{\delta }_{\textit{start}}}} \right)}}{{\left( {n - x} \right)\ \times \ \left[ {\textit{toluene}} \right] \times \ \left( {1 + {{R}_{\textit{VPDB}}}\ \times \ \left( {1 + {{\delta }_{\textit{start}}}} \right)} \right)\ }}\\
&&\quad \times \ 100.
\end{eqnarray*}

Equation 1 incorporates the δ values at the start and end of the experiment, estimated starting toluene concentrations based on the injection volume, and the variables n and x which represent the total and non-labelled carbon of the target compound, respectively. In this experiment, [^13^C]-α-toluene is the target substrate, with only single labelled carbon, hence *n* = 7 and *x* = 1. A previous study has shown that the methyl moiety of [^13^C]-α-toluene was better assimilated into the total lipid fatty acid (8–15%) compared to the fully labelled [^13^C_7_]-toluene (51–57%) (Bombach et al. 2010). Since the methyl moiety can end up as acetyl-CoA, precursors for fatty acids or carbon dioxide, [^13^C]-α-toluene is suitable to obtain mineralization data (Heider 2007, Lueders 2017).

The general equation for complete toluene mineralization and coupled sulfate reduction (linked to stoichiometric sulfide production) is as follows (Beller et al. 1992):


(2) \begin{eqnarray*}
{{{\mathrm{C}}}_{\mathrm{7}}}{{{\mathrm{H}}}_{\mathrm{8}}}{\mathrm{ + 4}}{\mathrm{.5SO}}_{\mathrm{4}}^{{\mathrm{2 - }}}{\mathrm{ + 3}}{{{\mathrm{H}}}_{\mathrm{2}}}{\mathrm{O}} \to {\mathrm{2}}{\mathrm{.25}}{{{\mathrm{H}}}_{\mathrm{2}}}{\mathrm{S + 2}}{\mathrm{.25H}}{{{\mathrm{S}}}^{\mathrm{ - }}}{\mathrm{ + 7HCO}}_{\mathrm{3}}^{\mathrm{ - }}{\mathrm{ + 0}}{\mathrm{.25}}{{{\mathrm{H}}}^{\mathrm{ + }}}.
\end{eqnarray*}

Toluene mineralization and sulfate reduction associated to cell growth can be described by (Beller et al. 1996)


(3) \begin{eqnarray*}
&&{{{\mathrm{C}}}_{\mathrm{7}}}{{{\mathrm{H}}}_{\mathrm{8}}}{\mathrm{ + 4}}{\mathrm{.09SO}}_{\mathrm{4}}^{{\mathrm{2 - }}}{\mathrm{ + 0}}{\mathrm{.17NH}}_{\mathrm{4}}^{\mathrm{ + }}{\mathrm{ + 2}}{\mathrm{.49}}{{{\mathrm{H}}}_{\mathrm{2}}}{\mathrm{O}} \to {\mathrm{2}}{\mathrm{.04}}{{{\mathrm{H}}}_{\mathrm{2}}}{\mathrm{S + 2}}{\mathrm{.04H}}{{{\mathrm{S}}}^{\mathrm{ - }}}\\
&&\qquad {\mathrm{ + 0}}{\mathrm{.17}}{{{\mathrm{C}}}_{\mathrm{5}}}{{{\mathrm{H}}}_{\mathrm{7}}}{{{\mathrm{O}}}_{\mathrm{2}}}{\mathrm{N\ (\textit{cells})\ + 6}}{\mathrm{.16HCO}}_{\mathrm{3}}^{\mathrm{ - }}{\mathrm{ + 0}}{\mathrm{.2}}{{{\mathrm{H}}}^{\mathrm{ + }}}.
\end{eqnarray*}

#### Sulfide analysis

Sulfide was quantified similarly to our previous studies via the modified methylene blue method described elsewhere (Cline 1969, Müller et al. 2009, Bin Hudari et al. 2022). Samples (50–200 µL) were collected over the course of the experiment via sterile N_2_-flushed syringes, added immediately to 1 mL of 3% zinc acetate dihydrate in glass test tubes followed by the consecutive addition of 4 mL bi-distilled water and 400 µL Cline reagent prepared as previously reported (Bin Hudari et al. 2022). After reagent addition, the mixture was mixed briefly, incubated in the dark for 20 min, mixed again and transferred to 2.5 mL cuvettes for absorbance measurement at 670 nm using a spectrophotometer (Novaspec III, Amersham Biosciences, Cambridge, United Kingdom) against a blank reference made of bi-distilled water.

### Microbial community analysis

Microcosms were sacrificed for microbial community analyses either at the end of the incubation period or if ^13^CO_2_ or sulfide production data for the replicate suggested that activities had ceased. Cells were harvested from solid materials (gravel or sediment) via a series of sodium tetrapyrophosphate modified treatment protocol as described elsewhere (Bin Hudari et al. 2022). Upon de-capping, ∼0.6–0.8 mL of sterile cold 1% sodium tetrapyrophospate (Sigma-Aldrich, USA) was added to each microcosm to a final concentration of ∼0.01% (v/v) and sonicated for three pulses of 10 s in an ice-water sonicating bath (Bandelin, Germany). The liquid fraction was poured into new pre-labelled 50 mL Falcon tubes. Five mL of 1% sodium tetrapyropshophate was added to the sediment and subjected to three pulses of 10 s of sonication in ice-water bath, and the liquid fraction was transferred to Falcon tubes. This step was repeated twice at 20-s pulses followed by a final pulse of 60 s. After each run, the liquid was transferred to Falcon tubes and kept cool in an ice bath. Falcon tubes were then centrifuged at 11 000 rpm at 4°C (Type 5804 R, Eppendorf, Germany). The supernatant was discarded and the pellet was stored at −38°C until DNA extraction. Extraction was carried out with the DNEasy Powersoil Kit (Qiagen, Hilden, Germany) mainly as per the manufacturer's instructions. Prior to DNA extraction, the tubes were first allowed to thaw for at least 30 min at 4°C, and the pellets were re-suspended with Powerbead Tube Buffer in the Falcon tubes before transfer, if necessary, more than one tubes were used per replicate. Samples were combined during the column spinning process to collect the precipitated DNA and finally eluted to ∼20–30 µL. Extracted DNA concentration was quantified with the Qubit HS (High Sensitivity) Assay Kit (Thermo Fisher Scientific, USA) on the Qubit 3.0 Fluorometer (Life Technologies, Malaysia). MiSeq sequencing procedures were similar to previous studies prescribed elsewhere (Bin Hudari et al. 2020, 2022) with the Klindworth primer pair (S-D-Bact-0341-b-S-17/S-D-Bact-0785-a-A-21) (Klindworth et al. 2013). Briefly, sequencing libraries were assembled with Illumina MiSeq Reagent Kit v3 (2 × 300 bp) as per manufacturer recommendations on 16S Metagenomic Sequencing Library Preparations (Illumina, 2013) and sequenced on the Illumina Miseq platform at the Department of Applied Microbial Ecology of the Helmholtz Centre for Environmental Research - UFZ. Sequences were analysed using QIIME 2 2025.4 (Bolyen et al. 2019) using a pipeline provided by Dr. Denny Popp. This pipeline includes removing primer sequences and adapters from the de-multiplexed sequences, trimming, and denoising to remove low-quality reads and chimeras, before merging. The silva138.2 database was used to assigned amplicon sequence variants (ASVs) to the bacterial DNA (Quast et al. 2013, Yilmaz et al. 2014). Prior to dissimilarity (α and ß) diversity analyses, samples were first rarefied to the number of reads of the sample with the least amount of sequences. Sequences were deposited at the European Nucleotide Archive (ENA) under the primary accession number PRJEB77513.

### Statistical analysis

To analyse the differences in median rates for Zt and Wg inocula at various temperatures, the toluene mineralization and sulfide production rates in Zt (*n* = 9) and Wg (*n* = 16) groups were tested using the Wilcoxon paired sample test with Bonferroni–Hochberg correction. A *P* value < 0.05 was considered statistically significant. All statistical analyses were done with the statistical computing software R (https://www.r-project.org/).

## Results

### Toluene mineralization and sulfide production at different temperatures and temperature change scenarios

Considerable sulfide production was observed in seven enrichment cultures: Zt12, Zt20, Zt25, Wg12, Wg25, Wg38, and Wg45; no cultures could be established at 60°C with both used materials, and at 38°C and 45°C with Zeitz gravel within prolonged (>1 year) incubation (data not shown).

#### Toluene mineralization and sulfide production rates at constant temperature

**Figure 1.**
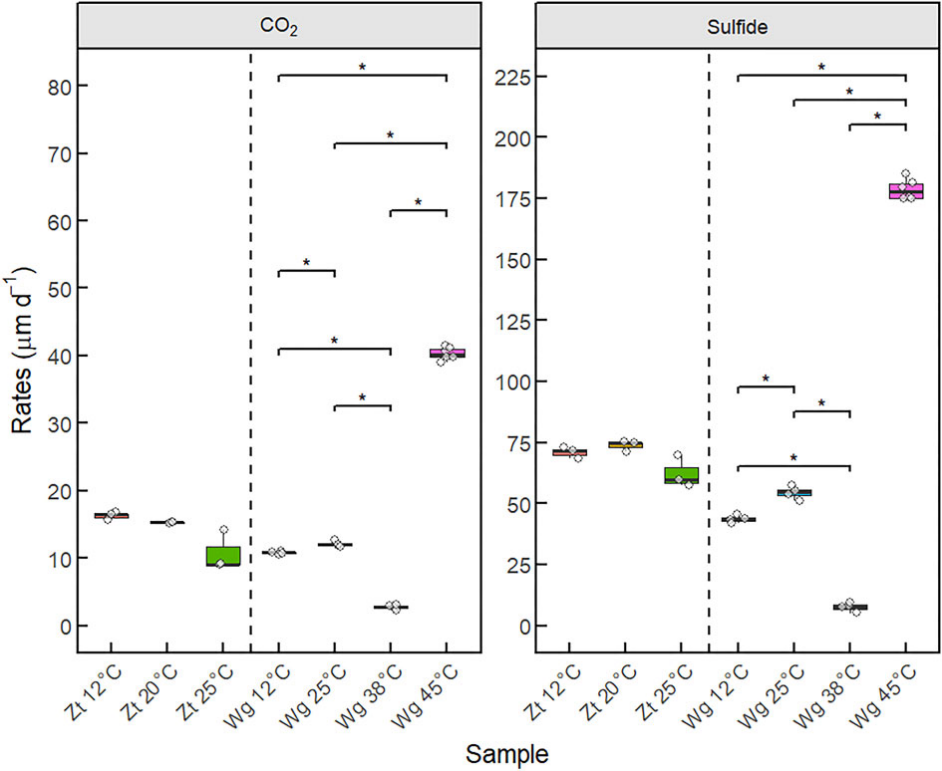
Boxplots of toluene mineralization and sulfide production rates, represented as average values (*n* ≥ 3). A Wilcoxon paired test was performed between the inocula with the sediment groups Zt and Wg, respectively. * indicates statistical significance (*P* ≤ 0.05).

[^13^C]-α-toluene mineralization and sulfide production rates of the seven setups (Zt12 to Wg45) are consolidated in Fig. 1 (for the complete data set, see Tables S8-S9_Supplementary Data and Figs S2-S5_Supplementary Data).

Microcosms incubated between 12 to 25°C (Zt12, Zt20, Zt25, Wg12 and Wg25) showed roughly comparable rates for toluene mineralization (10.7 ± 2.9–16.2 ± 0.6 µM d^−1^) and sulfide production (43.4 ± 1.5–73.6 ± 2.2 µM d^−1^). At 38°C (Wg38), toluene mineralization and sulfide production rates were around 4–6 and 6–10 folds lower, compared to the setups incubated at 12°C and 25°C, respectively, indicating non-optimal condition. The highest toluene mineralization and sulfide production rates were observed at 45°C (Wg45) that were around 2–4 folds (40.3 ± 0.9 and 178.5 ± 4.2 µM d^−1^) higher compared to the setups incubated at 12°C and 25°C, respectively. The pairwise Wilcoxon test indicated no significant differences (*P* > 0.05) in the median rates of both toluene mineralization and sulfide production between the Zt cultures at 12°C, 20°C, and 25°C. In contrast, significant differences (*P* ≤ 0.05, ‘*’) were observed in the median rates of toluene mineralization and sulfide production between the inoculum cultures at 12°C, 25°C, 38°C, and 45°C (Fig. 1).

Based on Equation (2), the expected ratio of sulfide produced to toluene mineralized is 4.5 in the case of complete mineralization. Of the seven ratio averages, five values were close to the expected ratio (4.0–4.8). Zt25 setups had the highest average ratio (6.0), whereas Wg38 had the lowest ratio of 2.7 (Table S9_Supplementary Data).

#### Effects of permanent temperature change on temperature-adapted cultures (‘adaptation’ scenario)

Active Zt12, Zt25, Wg12, WG25, and Wg38 cultures, firstly incubated at 12°C, 25°C, or 38°C for 28–34 days with [^13^C]-α-toluene, were transferred permanently to higher or lower temperatures for more than 100 days to determine immediate and long-term effects of temperature changes on toluene mineralization and sulfide production rates of the temperature-adapted enrichment cultures.

**Figure 2.**
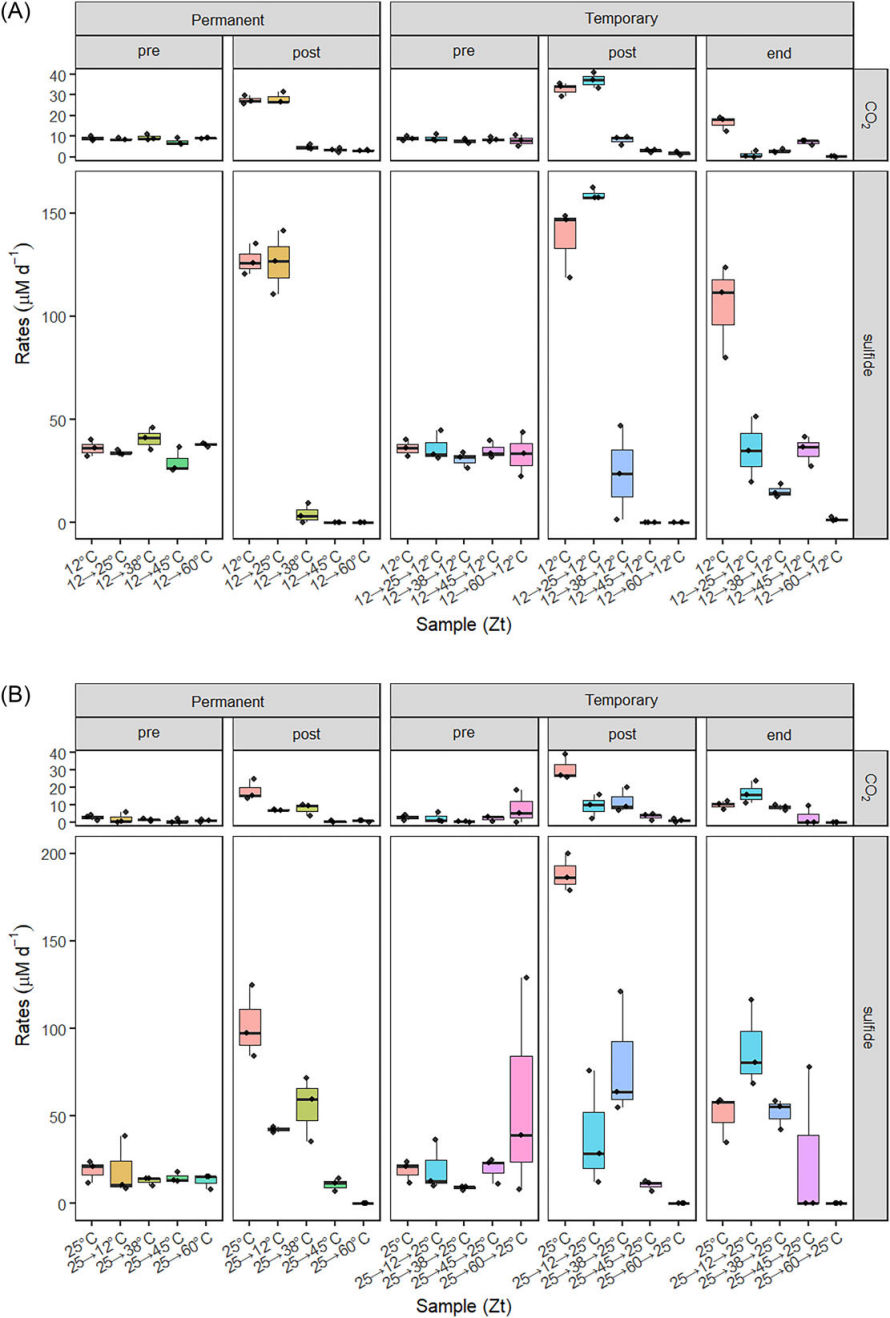
Rates of [^13^C]-α-toluene mineralization and sulfide production of Zt12 (A) and Zz25 (B) microcosms after permanent or temporary temperature change. Data are average values of replicate microcosms (*n* = 3). ‘Pre’ summarizes initial rates at 25°C. ‘Post’ summarizes rates after temperature change. ‘End’ summarizes rates after the temperature was changed back to the original temperature. For comparison, rates for microcosms incubated at constant temperature (12°C in A or 25°C in B) were calculated as well for each period. Data of single replicates and lengths of time periods are presented in Figs S6–S9 and S16–S19 and Tables S9, S10, and S12 of the Supplementary Data. Rates in 12→25→12 ‘end’ are low due to almost complete consumption of [^13^C]-α-toluene.

Zt12 setups switched to 25°C developed similarly as those incubated at 12°C (Fig. 2). When Zt12 cultures were moved to 38°C, toluene mineralization and sulfide production initially stopped but resumed at lower rates around 100 d after shifting to 38°C (Figs 2A; S6B, S7B_Supplementary Data). Zt12 cultures lost toluene mineralization activity when placed at 45°C or 60°C (Fig. 2A). Cultures adapted to 25°C (Zt25) showed lower toluene mineralization and sulfide production rates when shifted to 12°C and 38°C (Fig. 2B). At 38°C, toluene mineralization and sulfide production later ceased following prolonged incubation (Figs S8B, S9B_Supplementary Data). When shifted to 45°C and 60°C, Zt25 cultures mineralized toluene no longer (Fig. 2B).

**Figure 3.**
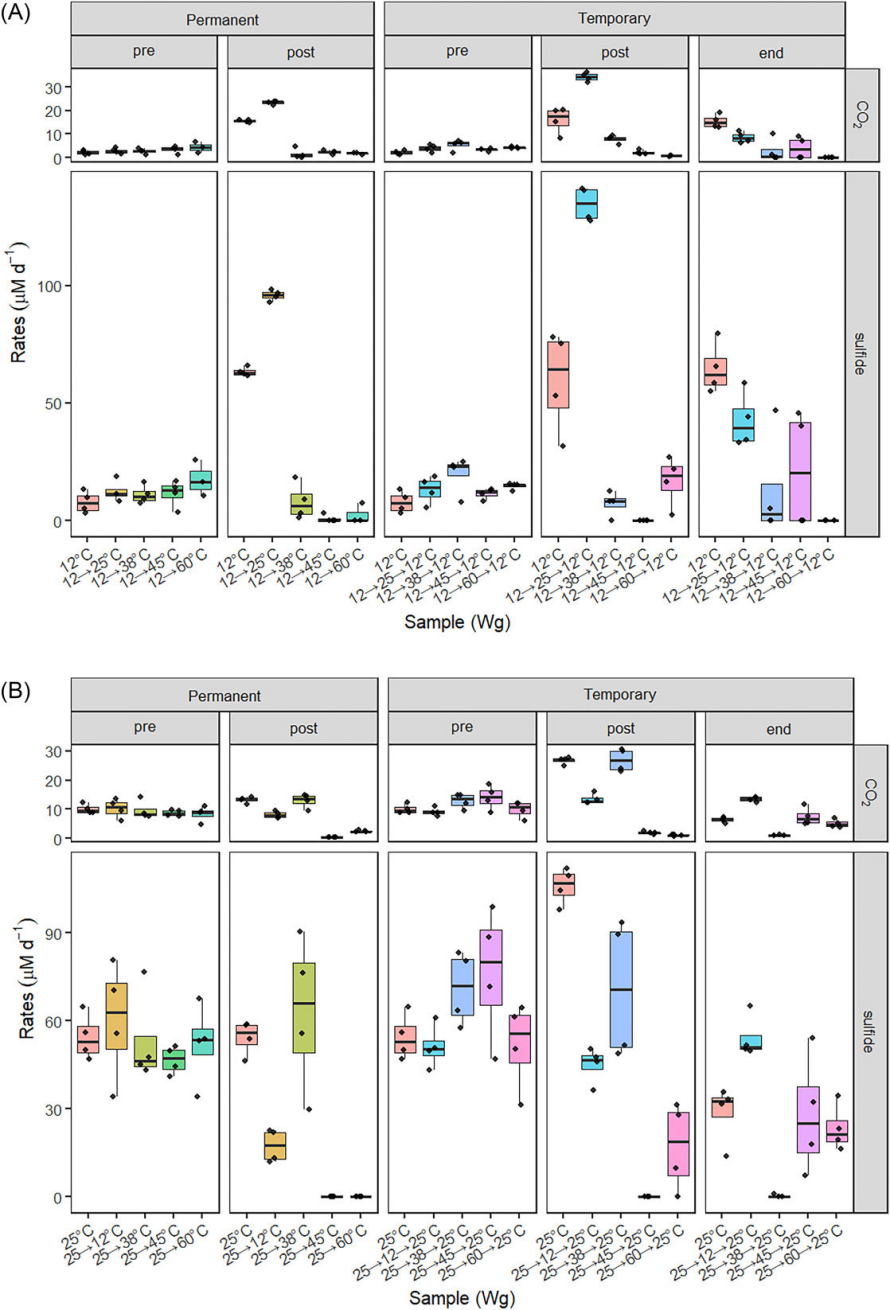
Rates of [^13^C]-α-toluene mineralization and sulfide production of Wg12 (A) and Wg25 (B) microcosms after permanent or temporary temperature change. Data are average values of replicate microcosms (*n* = 3). ‘Pre’ summarizes initial rates at 12°C (A) or 25°C (B). ‘Post’ summarizes rates after temperature change. ‘End’ summarizes rates after the temperature was changed back to the original temperature. For comparison, rates for microcosms incubated at constant temperature (12°C in A or 25°C in B) were calculated as well for each period. Data of single replicates and lengths of time periods are presented in Figs S10–S13 and S20-–S23 and Tables S9, S11, and S13 of the Supplementary Data.

Rates of Wg12 setups adapted at 12°C increased when transferred to 25°C while exposures to temperatures higher than 25°C almost completely inhibited toluene mineralization; only a single replicate exposed to 38°C showed sustained but lower toluene mineralization rates (Figs 3A, S10B_Supplementary Data). In Wg25 setups transferred from 25°C to 12°C, rates of toluene mineralization and sulfide production significantly decreased (Fig. 3B). In Wg25 setups transferred to 38°C, cultures stopped mineralizing toluene within 17 days (Fig. 12B_Supplementary Data); when exposed from 25°C to 45°C or 60°C, toluene mineralization ceased rapidly (Fig. 3B). Toluene mineralization and sulfide production rates at 38°C (Wg38) were generally considerably lower compared to cultures enriched at 12°C, 25°C, or 45°C (Fig. 1). When the cultures were moved to 25°C, activity was sustained but rates decreased (Figs 14B, 15B, 16_Supplementary Data). The Wg38 setups lost toluene mineralization activity when exposed to 12°C, 45°C, or 60°C (Figs S14, S15ACD, 16_Supplementary Data).

#### Effects of transient temperature change on temperature-adapted cultures (‘heat exchange scenario’)

Cultures Zt12, Zt25, Wg12, Wg25, and Wg38 were incubated for 28–34 days at 12°C, 25°C, or 38°C, respectively, then temporarily transferred to higher or lower temperatures for 14 days, and re-shifted to the initial incubation temperature.

Zt12 cultures incubated temporarily at 25°C preserved toluene mineralization and sulfide production rates (Fig. 2A). However, Zt12 cultures placed transiently at 38°C, 45°C, or 60°C lost toluene mineralization activity after temperature change (at 38°C, activity sustained for a couple of days); only cultures temporarily shifted to 38°C or 45°C began mineralizing toluene and producing sulfide 60–90 days after shifting back to 12°C (Figs 2A; S17, S18_Supplementary Data). In most Zt25 cultures transiently shifted to 12°C or 38°C, toluene mineralization and sulfide production rates decreased during the temperature shift phase (Fig. 2B) but activities regained when shifted back to 25°C (Figs 2B; S19AB, S20AB_Supplementary Data). Zt25 cultures transiently exposed to 45°C or 60°C lost toluene mineralization activity completely during the increased temperatures phase and afterwards, except for a replicate of the 25→45→25 approach that regained toluene mineralization and sulfide production activities more than 60 days after being returned to 25°C (Figs 2B; S19CD, S20CD_Supplementary Data).

Wg12 cultures mineralized toluene and produced sulfide at an accelerated rate when placed temporarily at 25°C (Fig. 3A). Higher temperatures (38°C, 45°C or 60°C) resulted in rapid or slightly delayed loss of toluene mineralization during exposure and, for a certain time, after resetting the temperature to 12°C (Figs 3A; S21BCD, S22BCD_Supplementary Data). Some of the 38°C and 45°C exposed replicates regained toluene mineralization activity more than 60 days after the temperature shift phase (Figs 21BCD, S22BCD_Supplementary Data). In Wg25 cultures, a temporary decrease in temperature to 12°C resulted in slightly lower toluene mineralization and sulfide production rates (Fig. 3B). In WG25 cultures temporary shifted to 38°C, toluene mineralization and sulfide production rates strongly decreased a couple of days after the shift to 38°C, and the rates remained at this low level for more than 100 days, until the end of incubation (Figs 3B; S23B, 24B_Supplementary Data). Wg25 cultures temporary shifted to 45°C or 60°C rapidly lost toluene mineralization activity and were inactive for 30 to up to more than 100 days after the temperature shift phase; then they started to mineralize toluene and produce sulfide again, in considerably higher rates than the replicates incubated temporarily at 38°C (Figs 3B; S23BCD, S24BCD_Supplementary Data). In Wg38 setups, a transient shift to 12°C, 25°C, 45°C, or 60°C inhibited toluene mineralization, but the cultures began mineralizing toluene and producing sulfide within 30 days after the temperature had been shifted back to 38°C. Notably, one replicate of the 12°C-affected culture showed higher toluene mineralization and sulfide production rates than observed before, and the 60°C-affected replicates mineralized toluene in lower rates compared to the non- and lower temperature affected 38°C replicates and did not produce sulfide (Figs S16, S25ABCA, S26ABC_Supplementary Data).

### Composition of toluene-mineralizing communities grown at different temperatures

#### Community composition of toluene-mineralizing communities enriched at constant temperature

**Figure 4.**
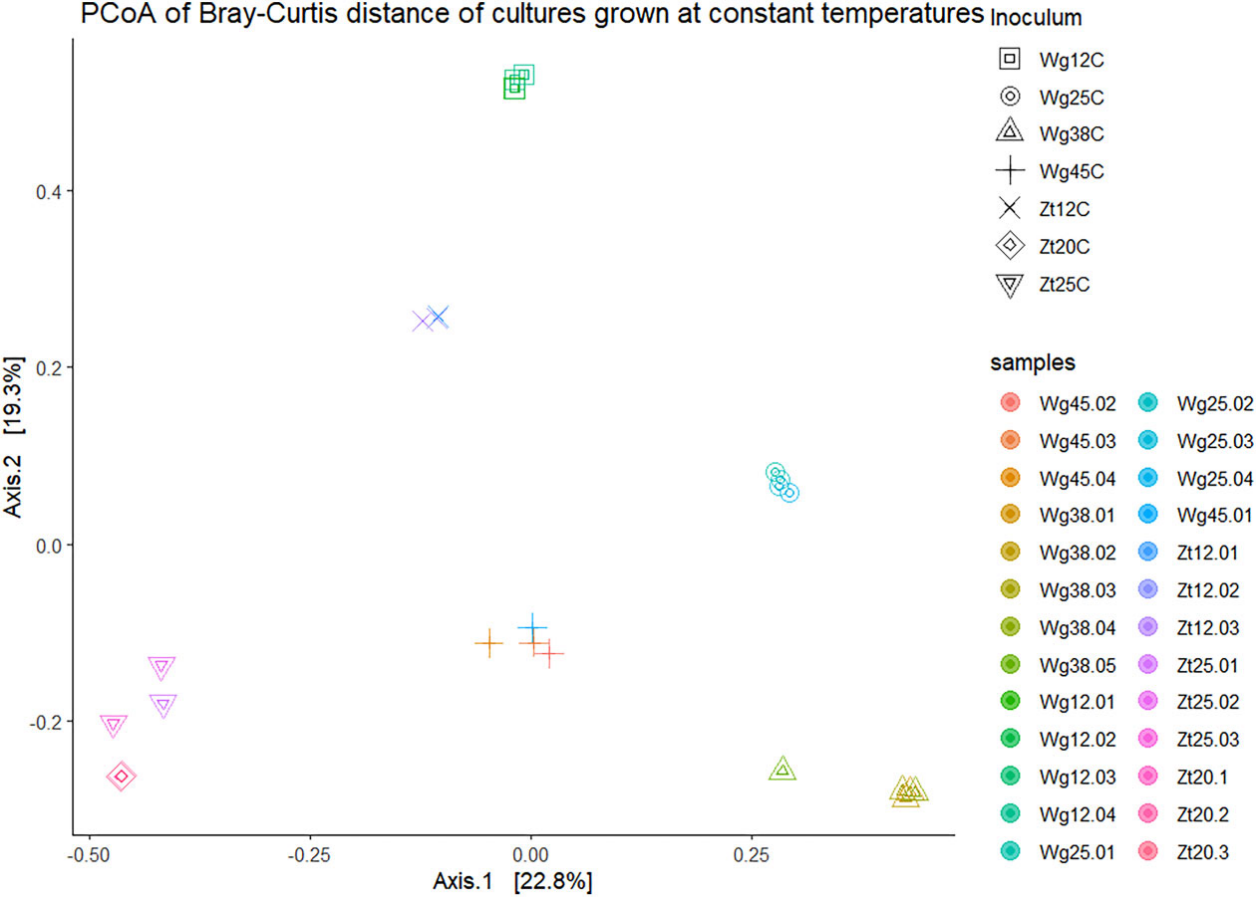
ß-diversity analysis according to BC dissimilarity of the cultures enriched with toluene and sulfate at various constant temperatures.

Distinct communities developed with regard to the selected temperature (12–45°C) and field site (Zt, Wg), as shown by analysis of β diversity (Fig. 4). The individual replicates were very similar to each other, indicating a high selection pressure for the community.

**Figure 5.**
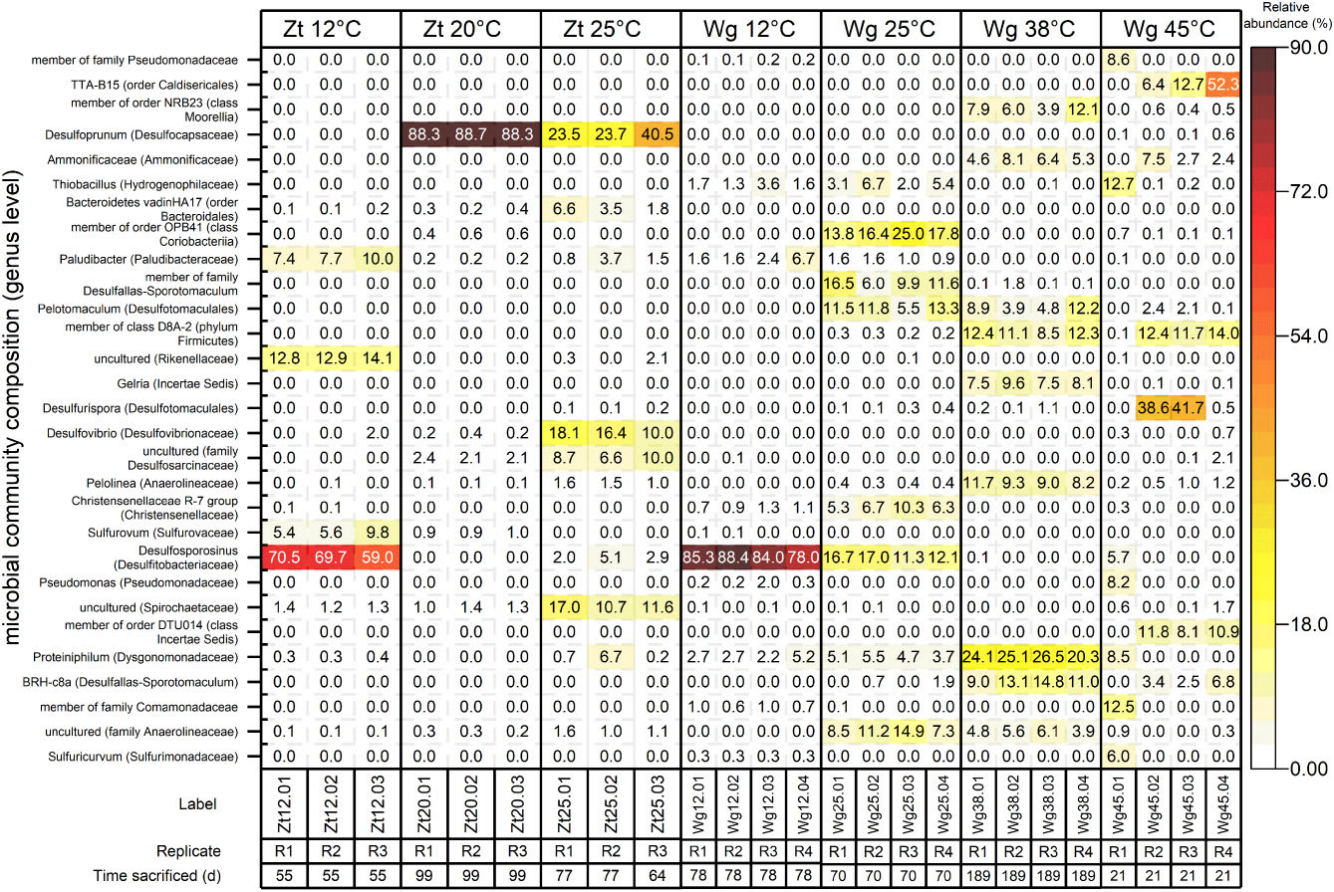
Microbial community composition of sulfate-reducing toluene-mineralizing Zt and Wg replicates incubated at different temperatures represented at the genus level and the corresponding family level in parentheses.

Microbial community compositions for the cultures enriched at constant temperatures (Zt12, Zt20, Zt25, Wg12, Wg25, Wg38, Wg45) are shown at the genera (Fig. 5) and family (Fig. S27_Supplementary Data) levels. At 12°C, phylotypes affiliated to the genus *Desulfosporosinus* (*Desulfitobacteriaceae*) were highly abundant in Zt12 (59%–71%) and Wg12 (78–88%) replicates. *Desulfosporosinus* phylotypes were less abundant at 25°C (2%–5% in Zt25; 11%–17% in Wg25) and negligible at 38°C and 45°C (Wg38, Wg45). At 20°C (Zt20), *Desulfoprunum* (88–89%) dominated in all replicate cultures. At 25°C, *Desulfoprunum* was also enriched in Zt25 (23%–41%), alongside *Desulfovibrio* (10–18%), a *Desulfosarcinaceae* affiliated phylotype (7%–10%) and organisms related to *Spirochaetaceae* (11%–17%). In Wg25, other phylotypes dominated compared to Zt25: *Pelotomaculum* (6%–13%), *Desulfallas-Sporotomaculum* (6%–16%), *Anaerolineaceae* (7%–15%), and organisms related to the order OPB41 (14%–25%) and the family *Christensenellaceae* (5%–10%). Different *Anaerolineaceae* (12%–17%), *Pelotomaculum* (4%–12%), and organisms belonging to *Desulfallas-Sporotomaculum* (9%–15%) were also found in Wg38 in higher abundances; in addition to phylotypes affiliated to *Ammonificaceae* (5%–8%), *Gelria* (8%–12%), the order NRB23 (4%–12%), class D8A-2 of the phylum *Bacillota* (9%–12%), and especially *Proteiniphilum* (20%–27%). The communities of Wg45 replicates differed from the communities enriched at lower temperatures and comprised mainly phylotypes related to TTA-B15 of the order *Caldisericales* (6%–52%), *Desulfurispora* (39%–42%, two replicates), the order DTU014 of the class *Incertae Sedis* (8%–12%), and the class D8A-2 of the phylum *Bacillota* (12%–14%) that were enriched in Wg38, too.

#### Community compositions in experiments with permanently changed temperatures (‘adaptation scenario’)

Generally, the cultures that were permanently exposed to a different temperature (higher or lower) underwent considerable community changes at temperature shifts from 12°C (or 25°C) to 38°C, 45°C, or 60°C as shown in the Bray–Curtis (BC) dissimilarity plots (Figs S28–S32_Supplementary Data). BC plots also show a change in the composition in Wg38 setups shifted to 12°C, 25°C, and 45°C with all the exposures clustered separately from setups maintained at constant temperature (Fig. S32_Supplementary Data).

In Zt12, the abundance of *Desulfosporosinus* phylotypes decreased when exposed permanently to 25°C (12%–17%) or 38°C (30%–36%) when compared to 12°C (59%–71%); instead, *Sulfurovum* (7%–19% at 25°C and 28%–38% at 38%) and uncultured *Rikenellaceae* (43%–64% at 25°C) became dominant (Fig. S33_Supplementary Data). In Zt12 cultures shifted permanently to 45°C and 60°C and not any longer mineralizing toluene, *Desulfosporosinus* phylotypes remained in high numbers (24%–53% at 45°C, 80%–92% at 60°C). In Zt25 setups shifted to 12°C, *Desulfosporosinus* abundances increased (10%–31%), while *Desulfovibrio* (12%–20%) and *Desulfoprunum* (8%–32%) persisted (Fig. S34_Supplementary Data). In Zt25 cultures shifted permanently to 38°C, uncultured members of the *Desulfosarcinaceae* (28%–30%) and *Spirochaetaceae* (20%–24%) became dominant; however, Zt25 cultures permanently shifted to 45°C and 60°C still contained phylotypes belonging to the genera *Desulfoprunum, Desulfosporosinus, Desulfovibrio* in higher abundances (20%–24%), although toluene mineralization coupled to sulfate reduction was inhibited.

In Wg12 cultures shifted to 25°C, *Desulfosporosinus* phylotypes were slightly less dominant (59%–70%) than observed at 12°C (77%–88%), and almost disappeared during incubations at 38°C and 45°C (0.4–6%); here, *Pelotomaculum* (at 38°C), *Proteiniphilum* (38°C, 45°C), *Thiobacillus* (38°C, 45°C), *Pseudomonas* (38°C), and *Anaerolineaceae* (38°C, 45°C) became dominant (Fig. S35_Supplementary Data). When shifted to 60°C, *Desulfosporosinus* remained in high numbers (51%–84%). In Wg25 cultures shifted to 12°C, *Desulfosporosinus* became more abundant (43%–45%) but was almost absent in cultures shifted to 38°C or 45°C; here, *Desulfitibacter* (at 38°C and especially at 45°C) and *Anaerolineaceae* (38°C, 45°C) became more abundant despite limited or absent toluene mineralization and sulfide production activity (Fig. S36_Supplementary Data).

In Wg38 cultures, members of the *Ammonificaceae* and the genus BRH-c8a (*Desulfallas-Sporotomaculum*) were still abundant when placed at 12°C, 45°C, or 60°C (Fig. S37_Supplementary Data), although setups shifted to these temperatures lacked toluene mineralization activity. *Proteiniphilum* were detected in higher numbers only at 12°C and 25°C, and members of the order NRB23 (class *Moorellia*) were enriched only at 45°C and 60°C.

#### Community compositions in temporary heat change experiments

Only communities enriched at 12°C (Zt12, Wg12) were considered with regard to community changes after a transient temperature shift. Both Wg and Zt communities transiently shifted to 25°C, 38°C, 45°C, or 60°C stopped to mineralize toluene temporarily at temperatures > 25°C but resumed to mineralize toluene in most replicates except those incubated temporarily at 60°C (Figs 3, S15, S16, S19_Supplementary Data). Effects of temperature on community structures were most pronounced at 45°C and 60°C, as shown by the BC dissimilarities (Figs S38, S39_Supplementary Data). Notably, *Desulfosporosinus* phylotypes that dominated the 12°C enrichments (Fig. 5) were still highly abundant in almost all of the replicates of the Zt and Wg series (Figs S42-S43_Supplementary Data). Phylotypes that had developed considerably (compared to non-affected 12°C communities, Fig. 5) during or after temperature change were affiliated to *Sulfurovum* (Zt12-38-12), uncultured *Rikenellaceae* (Zt12-25-12), uncultured *Spirochaetaceae* (Zt12-45-12), *Proteiniphilum* (Wg at 25°C, 38°C and 45°C) and *Gracilibacter* (Wg12-60-12), and phylotypes that got lost were affiliated to *Rikenellaceae* (Zt at 38°C, 45°C, 60°C) and *Proteiniphilum* (Wg 12–60-12) (Figs S40, S41_Supplementary Data).

## Discussion

### Toluene mineralization at sulfate-reducing conditions at different temperatures: key players and limits

Our data show that toluene was mineralized by aquifer communities from two different sites at sulfate-reducing conditions at temperatures between 12°C and 45°C. Enrichment cultures made from Weißandt-Gölzau sediment had a wider active temperature range (12°C–45°C) than cultures made of the Zeitz site material (12°C–25°C). In most cultures, the ratio of produced sulfide to mineralized toluene was close to the expected value of 4.1–4.5 based on equations (1) and (2), confirming the suitability of [^13^C]-*α*-toluene in monitoring toluene assimilation and mineralization processes as previously suggested (Bombach et al. 2010). Data across all seven setups investigated in this study show that toluene-mineralizing communities were generally negatively impacted at 38°C: no enrichments could be established at this temperature for the Zt community, and the Wg community showed considerably reduced mineralization rates, indicating unfavorable growth conditions. This corresponds mainly to data from previous studies. Anoxically incubated microbial communities from hydrocarbon-contaminated soil degraded toluene at 22°C and 30°C, but not at 35°C and 40°C (Zeman et al. 2014). Most described sulfate-reducing toluene-mineralizing consortia have been cultivated at temperatures between 14°C and maximal 35°C (Beller et al. 1992, Müller et al. 2009, Bombach et al. 2010, Cupples 2011, Pilloni et al. 2011, Sun and Cupples 2012). Different mesophilic sulfate-reducing toluene-mineralizing pure strains for which the temperature range for growth was determined in more detail could not grow above 36°C (Harms et al. 1999, Morasch et al. 2004, Ommedal and Torsvik 2007), with the exception of *Desulfosporosinus youngiae* growing at up to 39°C (Lee et al. 2009). However, a community from Weißand-Gölzau sediment could be established mineralizing toluene at 45°C in high rates and observed for the first time at this temperature. This finding demonstrates the presence of toluene-mineralizing organisms adapted to temperatures above the usual upper temperature limit of mesophilic microorganisms and indicates a previously unknown potential of aquifer microbial communities to degrade hydrocarbons in the lower thermophilic temperature range. We can only speculate why this potential was obviously absent in material from the Zeitz site. The lag-phase for toluene mineralization at 45°C in the Wg sediment was extraordinary long (>1 year), which indicates that these organisms were very sparsely distributed in the used environmental samples. Similar patterns—irregular activity of organisms with certain degradation capacities in environmental samples—have been observed, e.g. for anaerobic benzene degraders (summarized in Vogt et al. 2011) or anaerobic MTBE degraders (Somsamak et al. 2001).

However, toluene-mineralizing sulfate-reducing cultures could not be established in our study at temperatures higher than 45°C, although such organisms have been once described to be present in deep hot oil reservoirs (Chen and Taylor 1997); hence, it is still an open question whether aquifer communities are able to mineralize aromatic hydrocarbons at thermophilic or hyperthermophilic conditions; e.g. in course of an HT-ATES operation.

#### Key toluene-mineralizing organisms at native temperature

At native groundwater temperature (12°C), phylotypes affiliated to the genus *Desulfosporosinus* became highly abundant in microbial communities from both tested sites (Zt and Wg). This indicates that *Desulfosporosinus* members metabolize toluene as a sole carbon source far more quickly or efficiently than other putative toluene-degrading taxa at this temperature, indicating a major role for toluene (and probably other aromatic hydrocarbons) mineralization in aquifers at psychrophilic (native) conditions. Previously described members of *Desulfosporosinus* grew optimally at temperatures between 25°C and 37°C but were also shown to be adapted to 12°C or even lower temperatures (Robertson et al. 2001, Ramamoorthy et al. 2006, Lee et al. 2009). *Desulfosporosinus* species have already been described as anaerobic sulfate-reducing toluene degraders: Besides the mentioned *Desulfosporosinus youngiae* (Lee et al. 2009), a few *Desulfosporosinus* phylotypes were identified or suggested as toluene-metabolizing organisms in sulfate-reducing microbial communities sampled from soil or aquifer sediments (Robertson et al. 2000, Alfreider and Vogt 2007, Winderl et al. 2010, Sun and Cupples 2012) or were shown to metabolize toluene in methanogenic communities (Winderl et al. 2010, Cupples 2011, Fowler et al. 2014, Laban et al. 2015).

#### Key toluene-mineralizing organisms at temperatures above native conditions (12°C)

Temperatures above 12°C caused strong shifts in the toluene-degrading sulfate-reducing microbial community (Fig. 4). At 20°C, *Desulfoprunum* phylotypes became dominant in addition to *Desulfosporosinus* phylotypes (Fig. 5). At 25°C, Zt and Wg cultures were dominated by phylotypes belonging to the taxa *Desulfoprunum, Desulfovibrio, Pelotomaculum, Desulfallas-Sporotomaculum*, and uncultured members of *Anaerolineaceae. Desulfoprunum* is a genus currently comprising one species, *Desulfoprunum benzolyticum*, a mesophilic sulfate reducer that grows between 20° and 37°C, using benzoic acid as carbon and energy source (Junghare and Schink 2015, Galushko and Kuever 2019). In recent studies, sessile *Desulfoprunum* phylotypes were shown to accumulate at the surface of toluene-amended adsorption material placed in anaerobic groundwater microcosms, indicating that they had metabolized toluene (Taylor et al. 2021, Johnson 2023). *Desulfovibrio* are non-spore forming mesophilic sulfate reducers growing optimally between 30°C and 38°C; these organisms have not yet been described for their capability to degrade toluene, although they are commonly found in marine or freshwater habitats at sulfate-reducing conditions (Pelz et al. 2001, Müller et al. 2009, Bombach et al. 2010, Jehmlich et al. 2010, Fowler et al. 2014, Kuever et al. 2015). *Desulfovibrio* species are physiologically versatile sulfate reducers with the ability to consume hydrogen very efficiently (Cord-Ruwisch et al. 1988, Bak and Pfennig 1991, Müller et al. 2009). It is postulated that they can utilize metabolites of toluene metabolism or dead biomass of toluene degraders at mesophilic conditions (Pelz et al. 2001, Bombach et al. 2010), which could also be their ecological niche in our study. *Pelotomaculum* are gram-positive mesophilic/thermophilic endospore formers capable of utilizing low molecular weight aromatics such as benzene (Rainey 2015, Dong et al. 2017). So far, only *Pelotomaculum* candidate BPL has the complete sulfate reduction pathway for benzene degradation; meanwhile, other phylotypes seemed unable to carry out dissimilatory sulfate reduction and couple syntrophically the fermentation of organic substrates with other organisms (Dong et al. 2017). It is believed that *Pelotomaculum* phylotypes initially attack and oxidize benzene at anoxic conditions, perhaps incompletely to acetate and dihydrogen, which are used by hydrogenotrophic and acetotrophic sulfate reducers (Phelps et al. 1998, Herrmann et al. 2010, Weelink et al. 2010, Gieg et al. 2014, Rainey 2015, Dong et al. 2017, Lueders 2017). *Desulfallas-Sporotomaculum* phylotypes are usually anaerobic endospore formers, fermenters or sulfate reducers, and thermophilic or moderately thermophilic; members of the genus *Desulfallas* were classified under the genus *Desulfotomaculum* until a few years ago (Watanabe et al. 2018). Aromatics-utilizing *Desulfallas-Sporotomaculum* phylotypes have been described (Braumann et al. 1998, Kuever et al. 1999).

Other phylotypes appearing in permanent temperature shift setups include uncultured members of the actinobacterial lineage OPB41and *Spirochaetaceae*. OPB41 bacteria are globally distributed in the subsurface, but only two isolates have been described yet; one of them, the mesophilic strain M08DHB^T^, reduces anaerobically sulfur compounds using 3,4-dihydroxybenzoic acid as carbon and energy source (Khomyakova et al. 2022). Uncultured *Spirochaetaceae* were reported as necromass feeders (Dong et al. 2018).

#### Toluene-mineralizing community at 45°C

The toluene-metabolizing sulfate-reducing community enriched from Wg sediment at 45°C was considerably different to the communities enriched at 38°C and lower temperatures (Fig. 4), suggesting it was composed of toluene-metabolizing organisms specifically adapted to 45°C. Notably, different phylotypes had strongly accumulated (39%–52%) within replicates, affiliated to the genus *Desulfurispora* or the family TTA-B15 of the order *Caldisericales* (Fig. 5), indicating both may have used toluene as primary carbon source. *Desulfurispora* belongs to the order *Desulfotomaculales* in which spore-forming, often thermophilic sulfate reducers are classified (Chuvochina et al. 2023) which are colonizing the subsurface down to great depths (Aüllo et al. 2013); a few organisms belonging to this group have been reported as toluene degraders, too (Morasch et al. 2004, Lee et al. 2009). The family TTA-B15 of the order *Caldisericales* is less good known. Previous studies revealed that some members are sulfate reducers (Martinez et al. 2019) or associated with methanogenic crude oil degradation at thermophilic conditions (Cheng et al. 2014).

To our knowledge, anaerobic toluene mineralization at 45°C has not been reported so far. Degradation of other petroleum compounds (mainly alkanes) at thermophilic conditions by sulfate reducers has been shown in several studies (Rueter et al. 1994, Kniemeyer et al. 2007, Gieg et al. 2010, Mbadinga et al. 2012, Zhou et al. 2012, Khelifi et al. 2014). Dissimilatory sulfate reduction with other electron donors has been recorded at even higher temperatures, peaking at 110°C, carried out by microorganisms from a hydrothermal vent where rates ranged between 19 and 61 µM SO_4_^2−^ d^−1^ (Jørgensen et al. 1992). Sulfate-reducing laboratory cultures were successfully established at up to 85°C (Stetter et al. 1993, Marietou 2021). High sulfate reduction rates at temperatures > 50°C from up to 807 µmol L^−1^ to 5.3 ± 11 × 10^−10^ M s^−1^(∼45 792 µM d^−1^) were also reported (Gieg et al. 2010, Bonte et al. 2013, Li et al. 2017). Nonetheless, the mentioned studies with regard to anaerobic hydrocarbon degradation and dissimilatory sulfate reduction were conducted using material from areas naturally exposed to high temperatures and, hence, the native communities developed into thermophilic communities over long time periods by natural succession.

#### Impacts of temperature changes on toluene mineralization

Prolonged HT-ATES applications will result in temporarily or permanently lasting strong increases of temperature (‘heat shocks’) in distinct aquifer areas, either directly from close proximity to the core or in the further outer layers experiencing an gradual temperature increase (Daniilidis et al. 2022). In our study, the temporary exposure of heat was restricted to fourteen days to partially simulate seasonal temperature changes serving as a temporary heat shock, while the permanent transfer simulated consequences of areas with prolonged high temperature storage. Generally, temperature exposures ≥ 38°C were critical since toluene mineralization was severely limited or completely inhibited. These limitations would justify why the upper thresholds for LT-ATES are kept at 25°C (Fleuchaus et al. 2018), especially if combined with bioremediation. Perpetual absent sulfate reduction and toluene mineralization due to prolonged permanent incubation (>150 d) at 60°C indicates the absence of thermotolerant or thermophilic sulfate-reducing toluene-mineralizing community members operating at ≥ 60°C in our study. Notably, toluene-mineralizing communities enriched at *in situ* temperatures (12°C) exposed temporarily to 60°C could not restore its toluene-mineralizing activity, in contrast to the same communities temporarily exposed to 45°C (Fig. 3); thus, the higher the temperature, the more detrimental to the toluene-mineralizing community. Many *Desulfosporosinus* phylotypes can form heat-resistant endospores (Hippe and Stackebrandt 2015) allowing them to survive hostile environmental conditions, but it seems the toluene-mineralizing *Desulfosporosinus* of our study did not possess this property, at least not in the time frame of our experiments. Prolonged cyclic exposure at higher temperatures could hence generally damage the degradation potential of microbial communities of shallow aquifers, although this has to be investigated further, only a few studies investigating this topic are available (Yadav et al. 2012, Saito et al. 2016, Bin Hudari et al. 2022).

### Implications and outlook

This study highlights factors such as temperature limits, operational temperature, presence of thermophiles/thermotolerant or resilient ‘native’ communities in response to HT-ATES operations in hydrocarbon contaminated aquifers, using toluene as model contaminant and sulfate as electron acceptor. From a microbiological point of view, particularly the toluene (and generally aromatics hydrocarbon) mineralization potential above the temperature range of mesophilic organisms (≥45°C) is not understood but should in case of combining HT-ATES with bioremediation of hydrocarbon contaminated aquifers to be able to assess the consequences of the HT-ATES operation on the natural degradation potential of the aquifer. Hence, investigating the microbial community composition and remediation potential with regard to different temperatures should be addressed before trying to combine HT-ATES with bioremediation (Paliwal et al. 2012, Kaur et al. 2022). Currently, it remains unclear if anaerobic aromatics-degrading thermophiles or hyperthermophiles will be enriched by HT-ATES operations in shallow aquifers since these—as shown in our study—are seemingly absent or not cultivable at laboratory conditions at temperatures ≥ 60°C. Nevertheless, the enrichment of a specific community mineralizing toluene at 45°C from a shallow aquifer sediment sample that has never experienced temperatures above 15°C before hold some promise that aquifer sediments contain various different thermophiles having unexpected abilities and getting active at the right conditions and time; we are also aware that laboratory microcosm experiments can only provide limited evidence of processes that could take place *in situ* (Paliwal et al. 2012). Microcosms may differ from actual aquifers in substrate distribution and redox gradients, which can prevent a degradation potential observed in the laboratory from developing *in situ*. On the other hand, similar anaerobic hydrocarbon degradation reactions were shown to occur at laboratory, pilot and field scale (Fischer et al. 2009), demonstrating that processes observed in laboratory microcosms made of sediment samples may indeed reflect ongoing *in situ* processes.

Furthermore, studies must assess if continuous temperature change cycles can sustain activity, a resilient community and degradation potential, and if there will be a long-term thermal cycling fatigue. It is established that seasonal ATES can perturb the subsurface (Saito et al. 2016), but the effects of repeated heat exchange cycling at higher temperature thresholds as well as on nutrient availability and contaminants physicochemical characteristics need to be investigated (Head et al. 2014). For example, studies on organic carbon, total nitrogen or phosphorus changes in response to temperature have been described (Jiao et al. 2016, Geng et al. 2017, Gong et al. 2019). Future studies could incorporate multiple heat cycle experiments, or coupling different heat exchange scenarios with stable isotope probing to investigate the long-term effects of HT-ATES on ecosystem services and bioremediation. Looking forward, transitioning from LT-ATES to HT-ATES installation without compromising contaminant attenuation must be economically viable. Meanwhile, gaining societal ‘acceptance’ has to be conducted in parallel, perhaps by educating the masses of the long-term viability of HT-ATES and allaying concerns about ‘microbial desertification’. Otherwise, alternatives including bioaugmentation and biostimulation that provides either the essential communities or parameters to promote remediation can be introduced.

## Supplementary Material

fiaf079_Supplemental_Files

## Data Availability

All data are available on request, sequencing data have been deposited in a public data base as mentioned in the text.
