# Supplementary material for: Sulfidic toluene mineralization by aquifer microbial communities at different temperatures
Source: FEMS Microbiol Ecol. 2025 Jul 29;101(8):fiaf079. doi: 10.1093/femsec/fiaf079 (PMC12342453; doi:10.1093/femsec/fiaf079)
Supplement: fiaf079_Supplemental_Files [file fiaf079_supplemental_files.zip › Supplemental-Data-FiguresS1-11-BinHudari-revision.docx]

**Supplementary Data**

Sulfidic toluene mineralization by aquifer microbial communities at different temperatures

Mohammad Sufian Bin Hudari, Carsten Vogt*

Department of Technical Biogeochemistry, Helmholtz Centre for Environmental Research–UFZ, Leipzig, Germany

*Corresponding author:

[carsten.vogt@ufz.de](mailto:carsten.vogt@ufz.de)

Fon: +49 341 6025 1357

Figures **S1, S2, S3, S4, S5, S6, S7, S8, S9, S10, S11**

**Figure S1**. Overview how the enrichment cultures were set up and labeled and in which experiments they have been used.


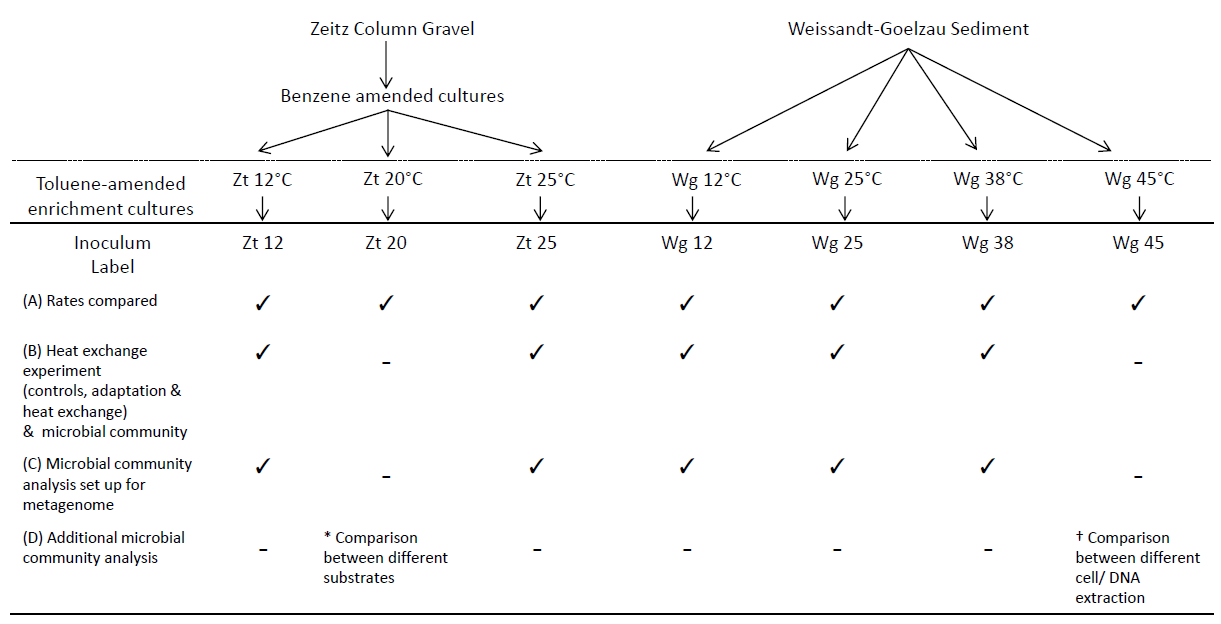


**Figure S2**. [^13^C]-α-toluene mineralization of Zt12, Zt20 and Zt25 setups permanently incubated at 12°C, 20°C and 25°C, respectively. Data are presented for each replicate and for sterilized controls.





**Figure S3**. Sulfide production of Zt12, Zt20 and Zt25 setups permanently incubated at 12°C, 20°C and 25°C, respectively. Data are presented for each replicate and for sterilized controls.





**Figure S4**. [^13^C]-α-toluene mineralization of Wg12, Wg25, Wg 38 and Wg45 setups permanently incubated at at 12°C, 25°C, 38°C or 45°C, respectively. Data are presented for each replicate and for sterilized controls.





**Figure S5**. Sulfide production of Wg12, Wg25, Wg 38 and Wg45 setups permanently incubated at at 12°C, 25°C, 38°C or 45°C, respectively. Data are presented for each replicate and for sterilized controls.





**Figure S6**. [^13^C]-α-toluene mineralization of Zt12 setups permanently incubated at higher temperatures: (A) Zt12→25°C (B) Zt12→38°C (C) Zt12→45°C (D) Zt12→60°C including the Zt12°C incubated at constant temperature (12°C), respectively. Data are presented for each replicate and the corresponding average of these replicates.





**Figure S7**. Sulfide production of Zt12 setups permanently incubated at higher temperatures: (A) Zt12→25°C (B) Zt12→38°C (C) Zt12→45°C (D) Zt12→60°C including the Zt12°C incubated at constant temperature (12°C), respectively. Data are presented for each replicate and the corresponding average of these replicates.





**Figure S8**. [^13^C]-α-toluene mineralization of Zt25 setups permanently incubated at higher temperatures: (A) Zt25→12°C (B) Zt25→38°C (C) Zt25→45°C (D) Zt25→60°C including the Zt25°C incubated at constant temperature (25°C), respectively. Data are presented for each replicate and the corresponding average of these replicates.





**Figure S9**. Sulfide production of Zt25 setups permanently incubated at higher temperatures: (A) Zt25→12°C (B) Zt25→38°C (C) Zt25→45°C (D) Zt25→60°C including the Zt25°C incubated at constant temperature (25°C), respectively. Data are presented for each replicate and the corresponding average of these replicates.





**Figure S10**. [^13^C]-α-toluene mineralization of Wg12 setups permanently incubated at higher temperatures: (A) Wg12→25°C (B) Wg12→38°C (C) Wg12→45°C (D) Wg12→60°C including the Wg12°C incubated at constant temperature (12°C), respectively. Data are presented for each replicate and the corresponding average of these replicates.





**Figure S11**. Sulfide production of Wg12 setups permanently incubated at higher temperatures: (A) Wg12→25°C (B) Wg12→38°C (C) Wg12→45°C (D) Wg12→60°C including the Wg12°C incubated at constant temperature (12°C), respectively. Data are presented for each replicate and the corresponding average of these replicates.
