# Supplementary material for: Sulfidic toluene mineralization by aquifer microbial communities at different temperatures
Source: FEMS Microbiol Ecol. 2025 Jul 29;101(8):fiaf079. doi: 10.1093/femsec/fiaf079 (PMC12342453; doi:10.1093/femsec/fiaf079)
Supplement: fiaf079_Supplemental_Files [file fiaf079_supplemental_files.zip › Supplemental-Data-FiguresS12-26-BinHudari-revision.docx]

**Supplementary Data**

Sulfidic toluene mineralization by aquifer microbial communities at different temperatures

Mohammad Sufian Bin Hudari, Carsten Vogt*

Department of Technical Biogeochemistry, Helmholtz Centre for Environmental Research–UFZ, Leipzig, Germany

*Corresponding author:

[carsten.vogt@ufz.de](mailto:carsten.vogt@ufz.de)

Fon: +49 341 6025 1357

**Figures S12, S13, S14, S15, S16, S17, S18, S19, S20, S21, S22, S23, S24, S25, S26**

**Figure 12**. [^13^C]-α-toluene mineralization of Wg25 setups permanently incubated at higher temperatures: (A) Wg25→12°C (B) Wg25→38°C (C) Wg25→45°C (D) Wg25→60°C including the Wg25°C incubated at constant temperature (25°C), respectively. Data are presented for each replicate and the corresponding average of these replicates.





**Figure S13**. Sulfide production of Wg25 setups permanently incubated at higher temperatures: (A) Wg25→12°C (B) Wg25→38°C (C) Wg25→45°C (D) Wg25→60°C including the Wg25°C incubated at constant temperature (25°C), respectively. Data are presented for each replicate and the corresponding average of these replicates.





**Figure S14**. [^13^C]-α-toluene mineralization of Wg38 setups permanently incubated at higher temperatures: (A) Wg38→12°C (B) Wg38→25°C (C) Wg38→45°C (D) Wg38→60°C including the Wg38°C incubated at constant temperature (38°C), respectively. Data are presented for each replicate and the corresponding average of these replicates.





**Figure S15**. Sulfide production of Wg38 setups permanently incubated at higher temperatures: (A) Wg38→12°C (B) Wg38→25°C (C) Wg38→45°C (D) Wg38→60°C including the Wg38°C incubated at constant temperature (38°C), respectively. Data are presented for each replicate and the corresponding average of these replicates.





**Figure S16**. Rates of [^13^C]-α-toluene mineralization and sulfide production of Wg38 microcosms after permanent or temporary temperature change. Data are average values of replicate microcosms (*n* = 3). ‘Pre’ summarizes initial rates at 38°C. ‘Post’ summarizes rates after temperature change. ‘End’ summarizes rates after the temperature was changed back to the original temperature. For comparison, rates for microcosms incubated at constant temperature (38°C) were calculated as well for each period. Data of single replicates and lengths of time periods are presented in Figs. S10-S13 and S20-S23 and Tables S9, S11 and S13 of the Supplementary Data.


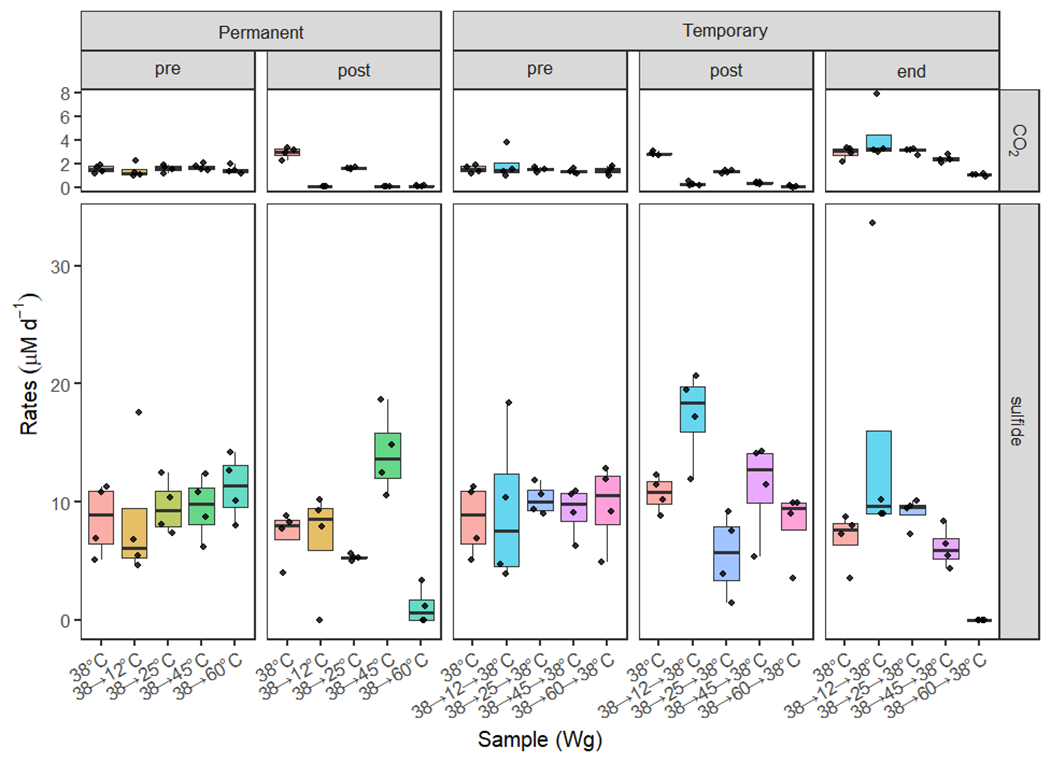


**Figure S17**. [^13^C]-α-toluene mineralization of Zt12 setups temporarily (14 d) incubated at higher temperatures: (A) Zt12→25→12°C (B) Zt12→38→12°C (C) Zt12→45→12°C (D) Zt12→60→12°C including replicates Zt12 incubated at constant temperature (12°C), respectively. Data are presented for each replicate and the corresponding average of these replicates.





**Figure S18**. Sulfide production of Zt12 setups temporarily (14 d) incubated at higher temperatures: (A) Zt12→25→12°C (B) Zt12→38→12°C (C) Zt12→45→12°C (D) Zt12→60→12°C including replicates Zt12 incubated at constant temperature (12°C), respectively. Data are presented for each replicate and the corresponding average of these replicates.





**Figure S19**. [^13^C]-α-toluene mineralization of Zt25 setups temporarily (14 d) incubated at higher temperatures: (A) Zt25→12→25°C (B) Zt25→38→25°C (C) Zt25→45→25°C (D) Zt25→60→25°C including replicates Zt25 incubated at constant temperature (25°C), respectively. Data are presented for each replicate and the corresponding average of these replicates.





**Figure S20**. Sulfide production of Zt25 setups temporarily (14 d) incubated at higher temperatures: (A) Zt25→12→25°C (B) Zt25→38→25°C (C) Zt25→45→25°C (D) Zt25→60→25°C including replicates Zt25 incubated at constant temperature (25°C), respectively. Data are presented for each replicate and the corresponding average of these replicates.





**Figure S21**. [^13^C]-α-toluene mineralization of Wg12 setups temporarily (14 d) incubated at higher temperatures: (A) Wg12→25→12°C (B) Wg12→38→12°C (C) Wg12→45→12°C (D) Wg12→60→12°C including replicates Wg12 incubated at constant temperature (12°C), respectively. Data are presented for each replicate and the corresponding average of these replicates.





**Figure S22**. Sulfide production of Wg12 setups temporarily (14 d) incubated at higher temperatures: (A) Wg12→25→12°C (B) Wg12→38→12°C (C) Wg12→45→12°C (D) Wg12→60→12°C including replicates Wg12 incubated at constant temperature (12°C), respectively. Data are presented for each replicate and the corresponding average of these replicates.





**Figure S23**. [^13^C]-α-toluene mineralization of Wg25 setups temporarily (14 d) incubated at higher temperatures: (A) Wg25→12→25°C (B) Wg25→38→25°C (C) Wg25→45→25°C (D) Wg25→60→25°C including replicates Wg25 incubated at constant temperature (25°C), respectively. Data are presented for each replicate and the corresponding average of these replicates.





**Figure S24**. Sulfide production of Wg25 setups temporarily (14 d) incubated at higher temperatures: (A) Wg25→12→25°C (B) Wg25→38→25°C (C) Wg25→45→25°C (D) Wg25→60→25°C including replicates Wg25 incubated at constant temperature (25°C), respectively. Data are presented for each replicate and the corresponding average of these replicates.





**Figure S25**. [^13^C]-α-toluene mineralization of Wg38 setups temporarily (14 d) incubated at higher temperatures: (A) Wg38→12→38°C (B) Wg38→25→38°C (C) Wg38→45→38°C (D) Wg38→60→38°C including replicates Wg38 incubated at constant temperature (38°C), respectively. Data are presented for each replicate and the corresponding average of these replicates.





**Figure S26**. Sulfide production of Wg38 setups temporarily (14 d) incubated at higher temperatures: (A) Wg38→12→38°C (B) Wg38→25→38°C (C) Wg38→45→38°C (D) Wg38→60→38°C including replicates Wg38 incubated at constant temperature (38°C), respectively. Data are presented for each replicate and the corresponding average of these replicates.
