# Supplementary material for: Sulfidic toluene mineralization by aquifer microbial communities at different temperatures
Source: FEMS Microbiol Ecol. 2025 Jul 29;101(8):fiaf079. doi: 10.1093/femsec/fiaf079 (PMC12342453; doi:10.1093/femsec/fiaf079)
Supplement: fiaf079_Supplemental_Files [file fiaf079_supplemental_files.zip › Supplemental-Data-FiguresS27-41-BinHudari-revision.docx]

**Supplementary Data**

Sulfidic toluene mineralization by aquifer microbial communities at different temperatures

Mohammad Sufian Bin Hudari, Carsten Vogt*

Department of Technical Biogeochemistry, Helmholtz Centre for Environmental Research–UFZ, Leipzig, Germany

*Corresponding author:

[carsten.vogt@ufz.de](mailto:carsten.vogt@ufz.de)

Fon: +49 341 6025 1357

Figures **S27, S28, S29, S30, S31, S32, S33, S34, S35, S36, S37, S38, S39, S40, S41**

**Figure S27**. Microbial community compositions of active Zt and Wg replicates incubated at different temperatures represented at the family level.





**Figure S28**. Principal component analysis (PCoA) of Bray-Curtis distance of Zt12 setups incubated at constant temperature of transferred permanently at higher temperature.


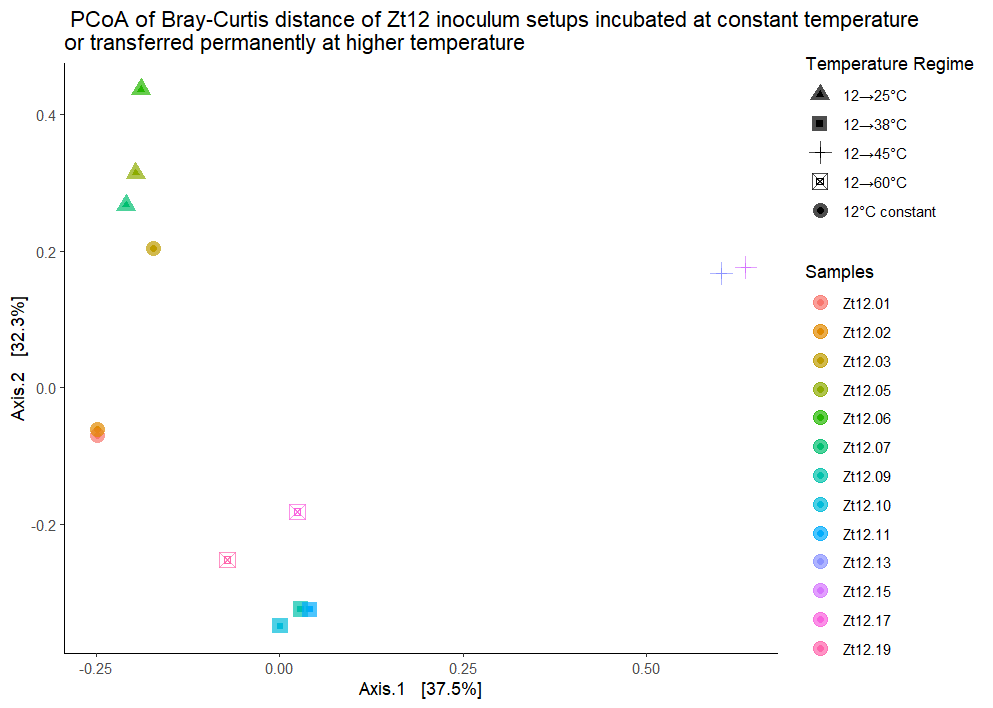


**Figure S29**. PCoA of Bray-Curtis distance of Zt25 setups incubated at constant temperature of transferred permanently at higher temperature.


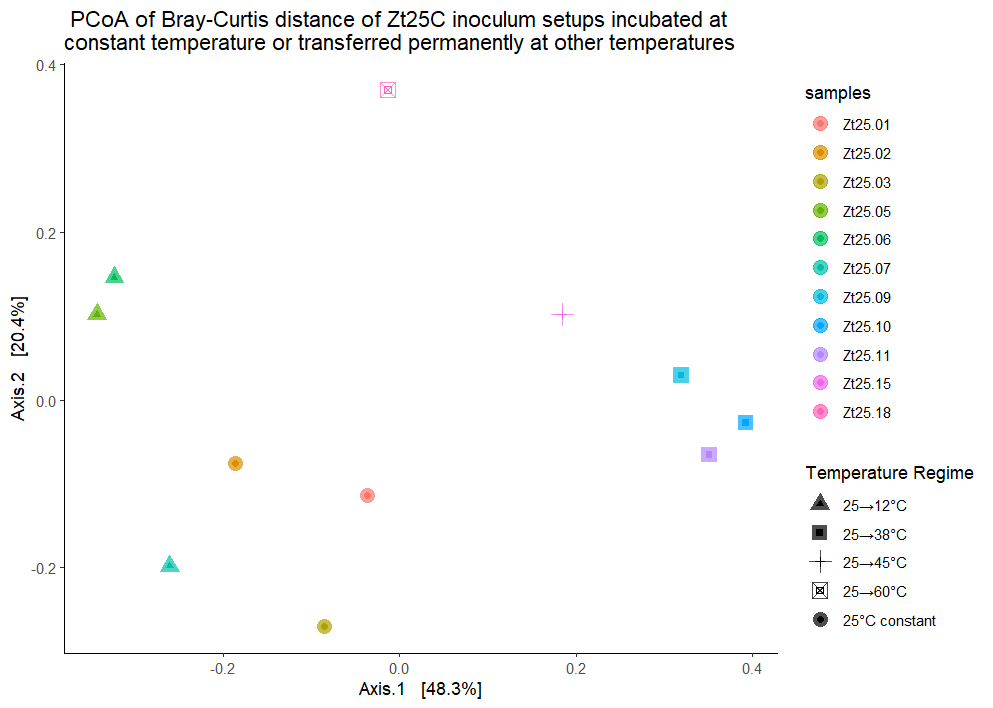


**Figure S30**. PCoA of Bray-Curtis distance of Wg12 setups incubated at constant temperature of transferred permanently at higher temperature.


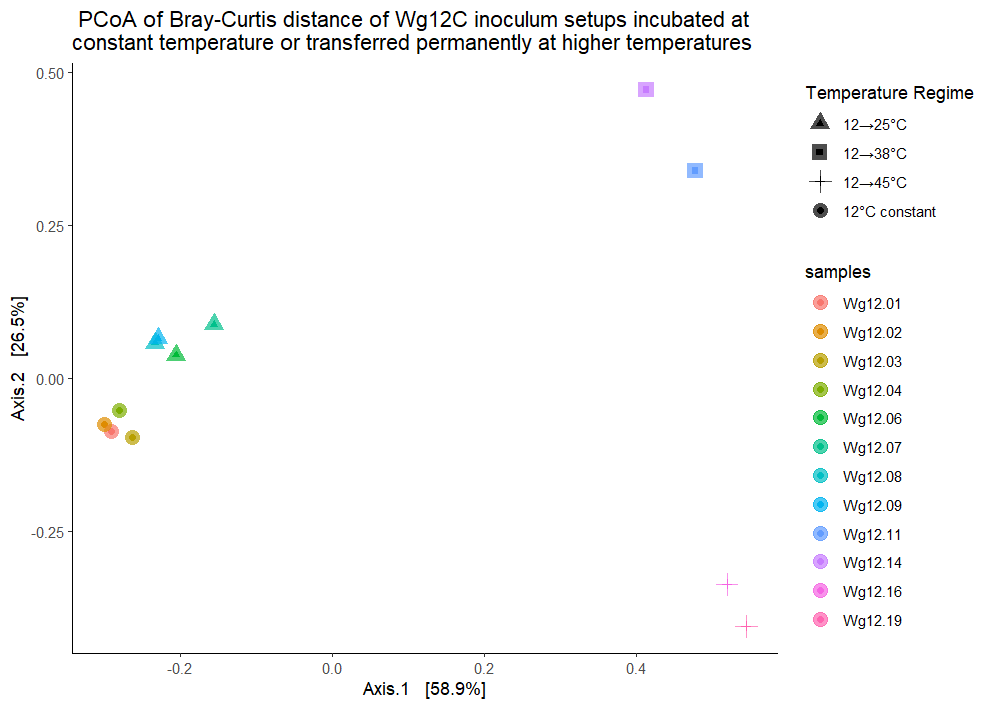


**Figure S31**. PCoA of Bray-Curtis distance of Wg25 setups incubated at constant temperature of transferred permanently at higher temperature.


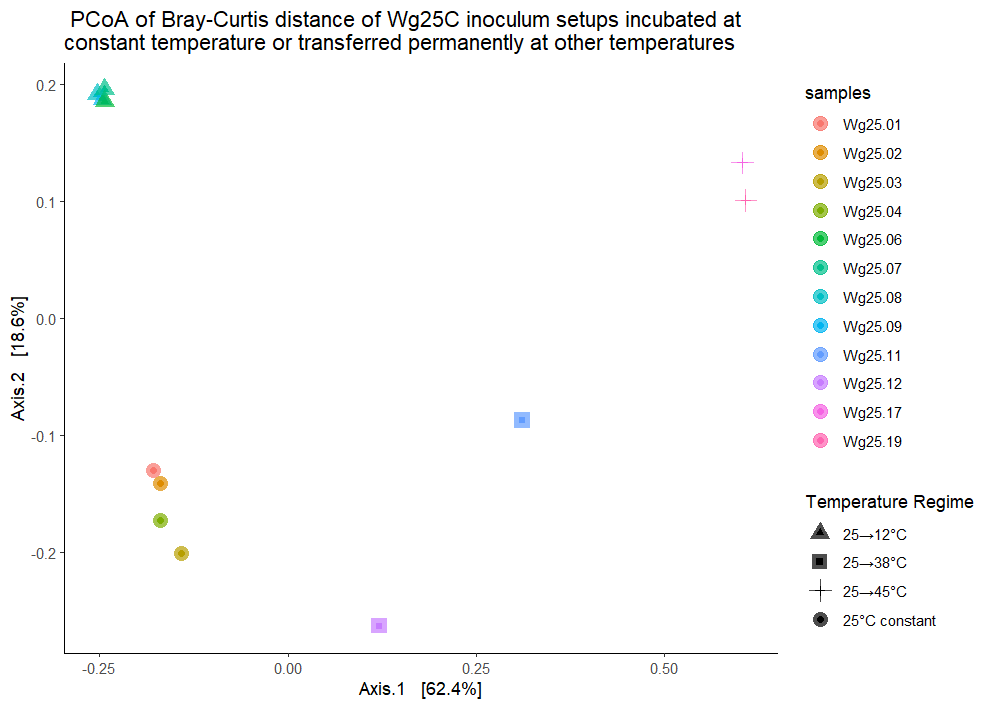


**Figure S32**. PCoA of Bray-Curtis distance of Wg38 setups incubated at constant temperature of transferred permanently at higher temperature.


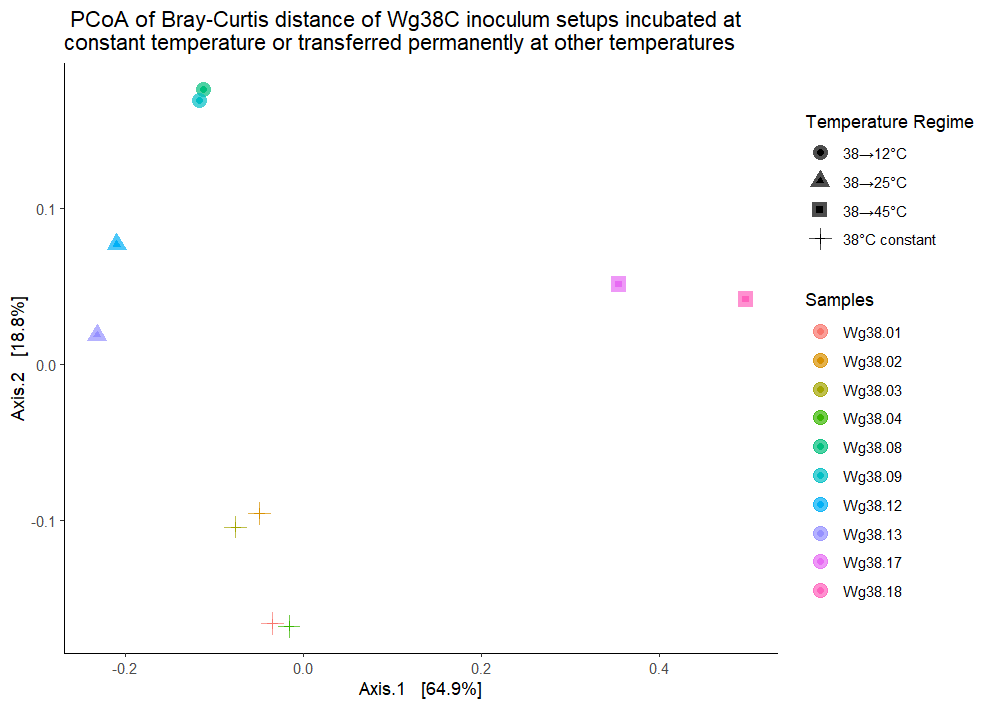


**Figure S33**. Microbial community compositions of active Zt12 replicates permanently shifted to higher temperatures.





**Figure S34**. Microbial community compositions of active Zt25 replicates permanently shifted to lower or higher temperatures.





**Figure S35**. Microbial community composition of active Wg12 replicates permanently shifted to higher temperatures.





**Figure S36**. Microbial community compositions of active Wg25 replicates permanently shifted to lower or higher temperatures.





**Figure S37**. Microbial community compositions of active Wg38 replicates permanently shifted to lower or higher temperatures.





**Figure S38**. PCoA of Bray-Curtis distance of Zt12 setups transiently incubated at higher temperatures (for 14 d).


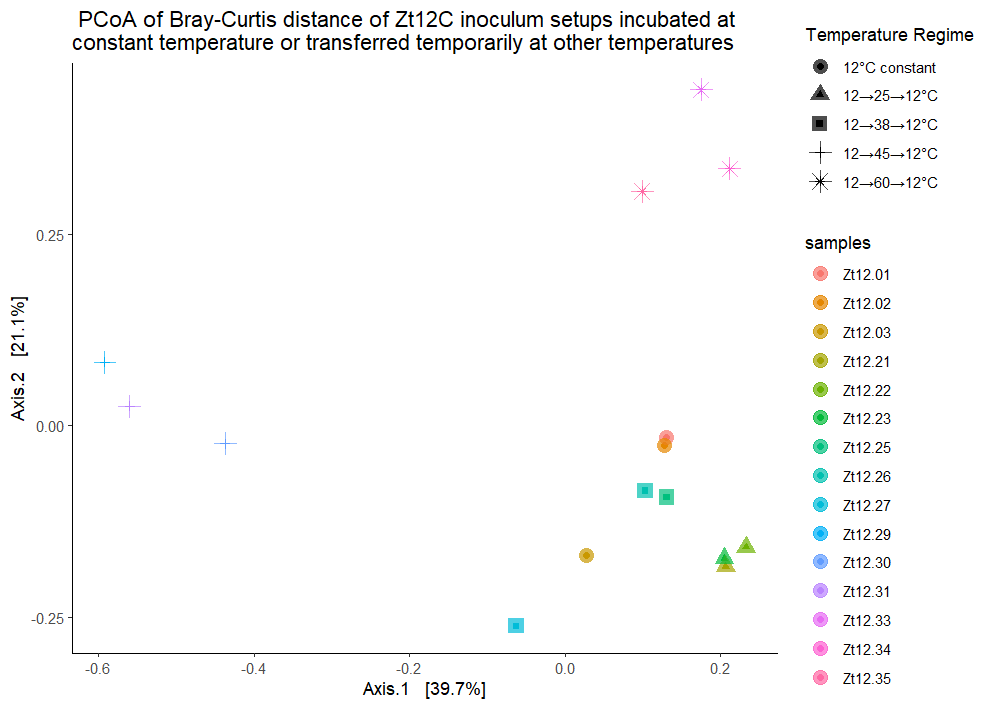


**Figure S39**. PCoA of Bray-Curtis distance of Wg12 setups transiently incubated at higher temperatures (for 14 d).


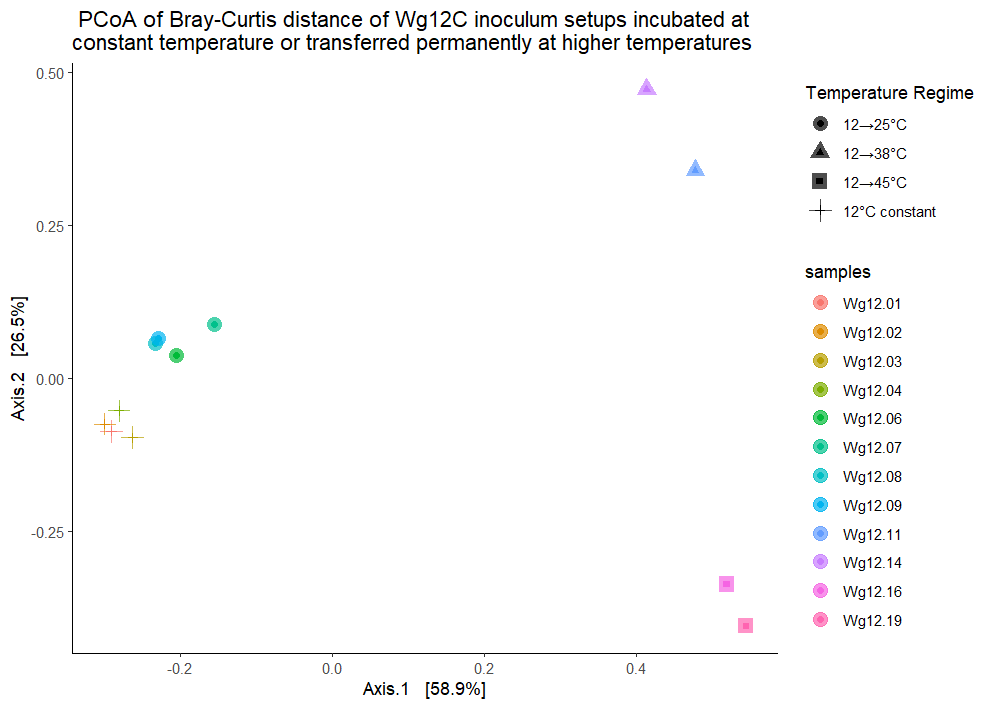


**Figure S40**. Microbial community compositions of active Zt12 replicates transiently shifted (14 d) to higher temperatures.





**Figure S41**. Microbial community compositions of active Wg12 replicates transiently shifted (14 d) to higher temperatures.
